# Kif13b Regulates PNS and CNS Myelination through the Dlg1 Scaffold

**DOI:** 10.1371/journal.pbio.1002440

**Published:** 2016-04-12

**Authors:** Roberta Noseda, Marta Guerrero-Valero, Valeria Alberizzi, Stefano C. Previtali, Diane L. Sherman, Marilena Palmisano, Richard L. Huganir, Klaus-Armin Nave, Ana Cuenda, Maria Laura Feltri, Peter J. Brophy, Alessandra Bolino

**Affiliations:** 1 Division of Neuroscience, INSPE-Institute of Experimental Neurology, San Raffaele Scientific Institute, Milan, Italy; 2 Department of Neurology, San Raffaele Scientific Institute, Milan, Italy; 3 Centre for Neuroregeneration, University of Edinburgh, Edinburgh, United Kingdom; 4 Hunter James Kelly Research Institute, Department of Biochemistry and Neurology, School of Medicine and Biomedical Sciences, State University of New York at Buffalo, Buffalo, New York, United States of America; 5 The Johns Hopkins University School of Medicine, Baltimore, Maryland, United States of America; 6 Department of Neurogenetics, Max Planck Institute of Experimental Medicine, Goettingen, Germany; 7 Department of Immunology and Oncology, Centro Nacional de Biotecnología/CSIC, Madrid, Spain; Stanford University School of Medicine, UNITED STATES

## Abstract

Microtubule-based kinesin motors have many cellular functions, including the transport of a variety of cargos. However, unconventional roles have recently emerged, and kinesins have also been reported to act as scaffolding proteins and signaling molecules. In this work, we further extend the notion of unconventional functions for kinesin motor proteins, and we propose that Kif13b kinesin acts as a signaling molecule regulating peripheral nervous system (PNS) and central nervous system (CNS) myelination. In this process, positive and negative signals must be tightly coordinated in time and space to orchestrate myelin biogenesis. Here, we report that in Schwann cells Kif13b positively regulates myelination by promoting p38γ mitogen-activated protein kinase (MAPK)-mediated phosphorylation and ubiquitination of Discs large 1 (Dlg1), a known brake on myelination, which downregulates the phosphatidylinositol 3-kinase (PI3K)/v-AKT murine thymoma viral oncogene homolog (AKT) pathway. Interestingly, Kif13b also negatively regulates Dlg1 stability in oligodendrocytes, in which Dlg1, in contrast to Schwann cells, enhances AKT activation and promotes myelination. Thus, our data indicate that Kif13b is a negative regulator of CNS myelination. In summary, we propose a novel function for the Kif13b kinesin in glial cells as a key component of the PI3K/AKT signaling pathway, which controls myelination in both PNS and CNS.

## Abstract 2

Kif13b is an unconventional kinesin that acts as a signaling molecule, regulating myelination via the Dlg1 scaffold in both Schwann cells (in the peripheral nervous system) and oligodendrocytes (in the central nervous system).

## Author Summary

Myelin is a multilayered extension of the Schwann and oligodendrocyte cell membranes, which wraps around neuronal axons to facilitate propagation of electric signals and to support axonal metabolism. However, the signals regulating myelin formation and how they are integrated and controlled to achieve homeostasis are still poorly understood.

In Schwann cells, the Discs large 1 (Dlg1) protein is a known brake of myelination, which negatively regulates the amount of myelin produced so that myelin thickness is proportional to axonal diameter. In this paper, we report that in Schwann cells Dlg1 itself is tightly regulated to ensure proper myelination. We propose that Dlg1 function is further controlled by the Kif13b kinesin motor protein, which acts as a "brake of the brake" by downregulating Dlg1 activity. Surprisingly, we found that in oligodendrocytes Dlg1 is a positive and not a negative regulator of myelination. Thus, Kif13b-mediated negative regulation of Dlg1 ensures appropriate myelin production and thickness in the central nervous system. Our data further extend recently emerged unconventional roles for kinesins, which are usually implicated in cargo transport rather than in the modulation of signaling pathways. The elucidation of mechanisms regulating myelination may help to design specific approaches to favor re-myelination in demyelinating disorders in which this process is severely impaired.

## Introduction

Myelination is a multistep process that includes axon recognition and contact, ensheathment, and myelin biogenesis. In this process, discrete sets of proteins and lipids are specifically assembled to generate and maintain distinct structural and functional domains necessary for nerve function [1–5]. During myelination, positive and negative regulators must be tightly controlled so that myelin thickness is strictly proportional to axonal diameters. However, the molecular mechanisms that promote and regulate myelination as well as the molecular machineries responsible for the transport and targeting of vesicles during myelin biogenesis are largely unknown. For example, Kif1b is the only motor protein identified thus far implicated in central nervous system (CNS) myelination in *Danio rerio* (zebrafish) [6].

We previously reported that in Schwann cells the Kif13b motor protein (also known as guanylate kinase-associated kinesin [GAKIN] in humans) is part of a complex that titrates membrane formation during Schwann cell myelination [7]. We found that Kif13b interacts with the Discs large 1 (Dlg1) scaffold in Schwann cells and that the downregulation of either *Kif13b* or *Dlg1* expression in Schwann cell/dorsal root ganglia (DRG) neuron co-cultures decreases myelination in vitro [7]. Another study independently reported that Dlg1-silenced Schwann cells in vitro showed migration defects and reduced expression of the polarity protein Par3 [8]. Occasionally, silenced cells overcame their migration defect and myelinated, but the resulting myelin segments were thicker than those of controls, which indicated Dlg1 as a negative regulator of myelin sheath thickness [8]. This role was further assessed in vivo, as we and others subsequently reported that mouse nerves lacking Dlg1 expression specifically in Schwann cells have hypermyelination, myelin outfoldings, and demyelination as a consequence of myelin instability [8,9]. Dlg1 is thought to act in complex with phosphatase and tensin homolog (PTEN) to reduce AKT (v-AKT murine thymoma viral oncogene homolog) activation; thus, it is a brake on myelination [8].

Kif13b kinesin is a plus end motor protein that mediates the transport of several cargos in polarized cells [10–16]. In PC12 cells, Kif13b negatively regulates centaurin-α_1_/PIP_3_BP (phosphatidylinositol-3,4,5-trisphosphate binding protein), a GTPase activating protein (GAP) for Arf6 (ADP-ribosylation factor) GTPase and promotes Arf6 plasma membrane activation [16]. In neurons, Kif13b transports centaurin-α_1_/PIP_3_BP and PIP_3_ to the tip of neurites to promote neuronal polarity [11].

To further investigate the function of the Kif13b/Dlg1 complex in myelination in vivo, we generated a novel *Kif13b* floxed allele and conditional knock-out mouse models with specific ablation of *Kif13b* or *Dlg1* in either Schwann cells or oligodendrocytes. Here, we report that Kif13b has opposite roles in the control of myelination in the peripheral nervous system (PNS) and CNS. Our data indicate that in Schwann cells, Kif13b interacts with p38γ mitogen-activated protein kinase (MAPK) to promote phosphorylation and ubiquitination of Dlg1. Consistent with this observation, loss of Kif13b results in reduced levels of p38γ MAPK, increased Dlg1 expression, and reduced myelin thickness.

Finally, we report that Kif13b also controls Dlg1 function in oligodendrocytes by promoting its negative regulation. However, our data indicate that, in contrast to Schwann cells, Dlg1 does not reduce but rather enhances AKT activation in oligodendrocytes. Thus, Kif13b is a novel negative regulator of CNS myelination.

## Results

### Kif13b Regulates Myelin Thickness in Schwann Cells

**Fig 1:**
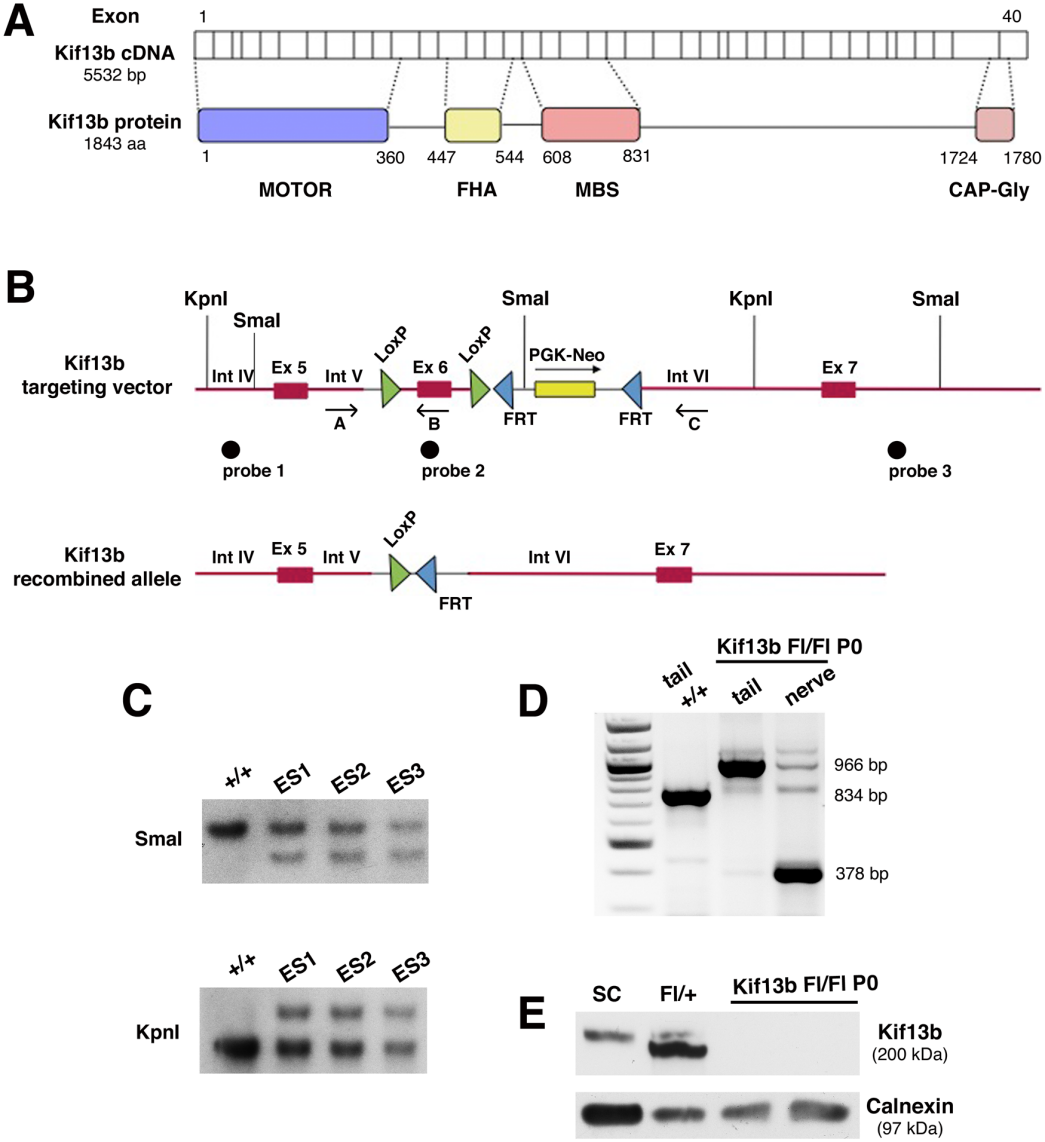
Generation of the *Kif13b-*null mouse in Schwann cells. (A) Kif13b protein domains. (B) The *Kif13b* targeting vector and the recombined allele following deletion of exon 6. Thick red lines represent genomic sequences, and thin black lines correspond to the pFlrt1 vector used for homologous recombination. (C) Southern blot analysis performed to identify ES cell recombinant clones. (D) PCR analysis using primers A and C performed on DNA from tail and sciatic nerve of wild type (+/+) and *Kif13b* ^*Fl/Fl*^ *P0-Cre* mice. (E) Western blot analysis on lysates from mouse sciatic nerves and isolated rat Schwann cells shows reduction of Kif13b expression in the mutant.

We previously reported that, in the peripheral nerve, Kif13b is mainly detected in cytosolic compartments of myelin-forming and non-myelin-forming Schwann cells [7]. To investigate the role of Kif13b in Schwann cells in vivo, we generated a *Kif13b* ^*Floxed*^ (hereafter, *Kif13b* ^*Fl*^) allele in which exon 6 was flanked by *lox-P* sites. Using the Cre/*loxP* technology, excision of exon 6 produces a frameshift leading to the introduction of a premature stop codon and to nonsense-mediated mRNA decay (Fig 1A–1C). To ablate *Kif13b* specifically in Schwann cells, we generated *Kif13b* ^*Fl/Fl*^ *P0-Cre* mice, in which the myelin protein zero (*MPZ*) promoter drives Cre recombinase expression specifically in Schwann cells, starting from E13.5 [17,18]. Deletion of exon 6 was documented by PCR analysis on DNA from the sciatic nerve, where a recombination band of 378 bp was specifically detected (Fig 1D). Kif13b protein expression was ablated in sciatic nerve lysates from *Kif13b* ^*Fl/Fl*^ *P0-Cre* mice, thus also confirming that Kif13b is mainly expressed by Schwann cells in the nerve (Fig 1E).

**Fig 2:**
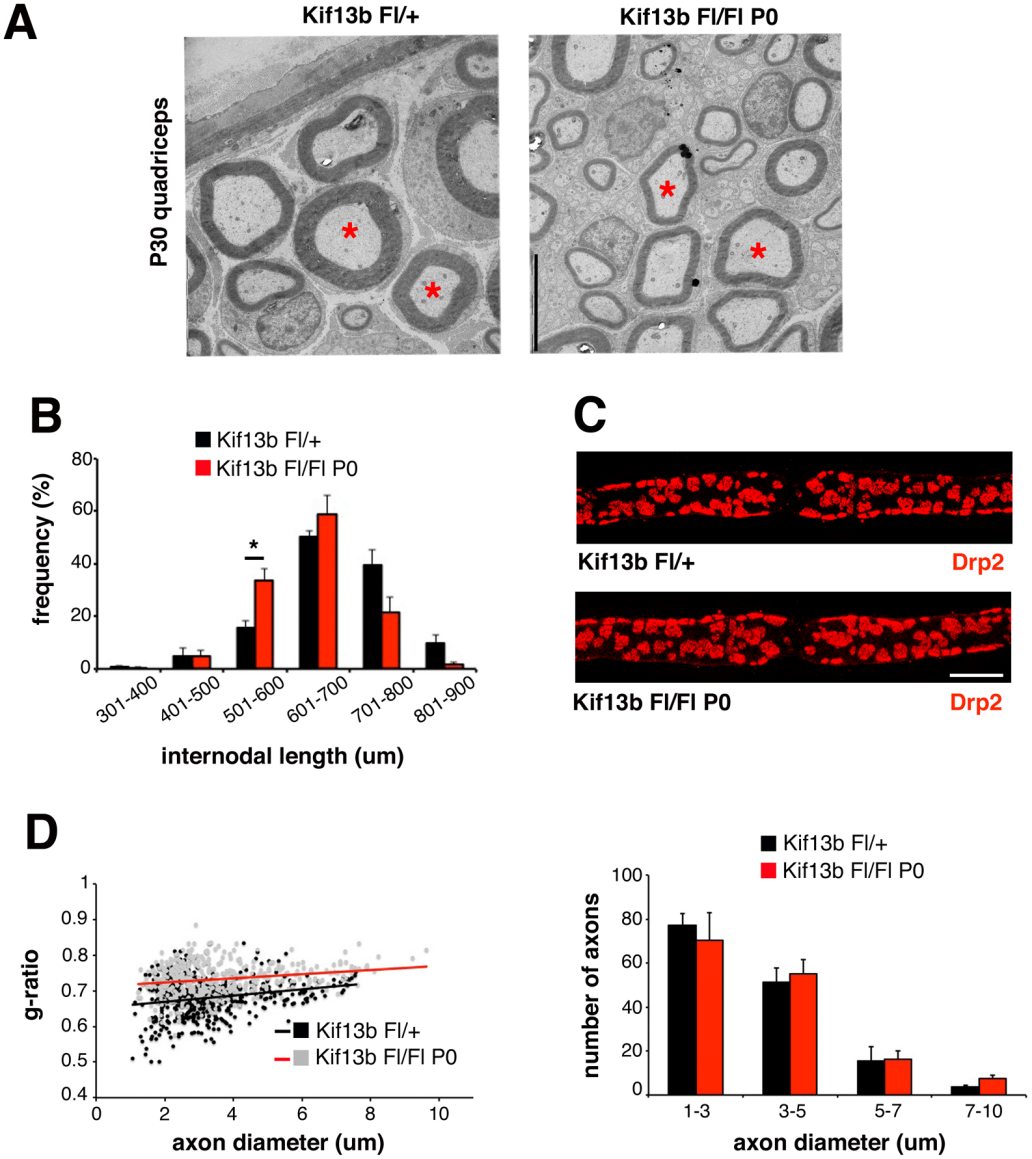
Kif13b regulates myelin thickness in Schwann cells. (A) Ultrastructural analysis of quadriceps nerves at P30. Red asterisks indicate thinner myelinated fibers in the mutants as compared to control fibers with similar axonal diameter. (B) Analysis of internodal length in quadriceps nerves at P30. The percentage (%) of fibers in the range 501–600 μm is 15.7 ± 2.61 for *Kif13b* ^*Fl/+*^ and 33.7 ± 4.26 for *Kif13b* ^*Fl/Fl*^ *P0-Cre*. *n* = 3 animals per genotype, *p* = 0.031. (C) Immunohistochemistry using anti-Drp2 antibody on teased fibers shows that Cajal bands structure is preserved in *Kif13b* ^*Fl/Fl*^ *P0-Cre* quadriceps nerves. (D) Quantification of the g-ratio as a function of axonal diameter (*Kif13b* ^*Fl/Fl*^ *P0-Cre*, 0.73 ± 0.006, 447 fibers; *Kif13b* ^*Fl/+*^, 0.68 ± 0.005, 443 fibers, *n* = 3 animals per genotype, *p* = 0.003) and axonal diameter distribution at 8 mo, ultrastructural analysis. The total number of fibers was normal. Bar in (A) and (C) is 5 μm.

We then analyzed *Kif13b* ^*Fl/Fl*^ *P0-Cre* sciatic nerves starting at P30 by performing semithin section and ultrastructural analyses. In mutant nerves, we noted a higher number of fibers displaying Schwann cell nuclei and the surrounding cytoplasm, suggesting a shorter internodal length (Fig 2A). Consistent with this, we found that *Kif13b* ^*Fl/Fl*^ *P0-Cre* quadriceps nerves had indeed a higher percentage of fibers with shorter internodes, particularly in the range between 500 and 600 μm (Fig 2B). Cajal bands are cytoplasmic channels located at the abaxonal surface of myelinated fibers and are involved in the biosynthesis and assembly of myelin [3]. Ablation of the Schwann cell protein Periaxin disrupts Cajal bands and is also associated with reduced longitudinal growth of Schwann cells [19]. However, subsequent work from the same group has shown that loss of Cajal bands as a result of Drp2 ablation causes focal hypermyelination and concomitant demyelination [20]. We analyzed *Kif13b* ^*Fl/Fl*^ *P0-Cre* quadriceps nerves, but we did not detect major differences in Cajal band structures between mutant and control nerves (Fig 2C). Our findings are consistent with the view that the longitudinal growth of Schwann cells does not correlate with Cajal band integrity [20]. As myelin thickness is proportional to axonal diameter and internodal length [3], we evaluated myelin thickness in *Kif13b* ^*Fl/Fl*^ *P0-Cre* nerves by performing ultrastructural analysis. By measuring the g-ratio—the ratio between axonal diameter and fiber diameter—we observed reduced myelin thickness in mutant quadriceps nerves at P30, which displayed increased g-ratio values as compared to controls (ultrastructural analysis, *Kif13b* ^*Fl/Fl*^ *P0-Cre*, 0.75 ± 0.008, 575 fibers; *Kif13b* ^*Fl/+*^, 0.70 ± 0.009, 516 fibers, *n* = 3 animals per genotype, *p* = 0.03). At P20, myelin thickness was normal in *Kif13b* ^*Fl/Fl*^ *P0-Cre* nerves, suggesting that myelination is not delayed in this mutant (ultrastructural analysis, g-ratio values: *Kif13b* ^*Fl/Fl*^ *P0-Cre*, 0.715 ± 0.007, 400 fibers; *Kif13b* ^*Fl/+*^, 0.71 ± 0.004, 403 fibers, *n* = 3 animals per genotype, *p* = 0.49). Reduced myelin thickness was still present in nerves of older mice at 8 mo, as g-ratio values were increased in mutant nerves (Fig 2D).

Finally, following crush nerve injury, remyelinating *Kif13b* ^*Fl/Fl*^ *P0-Cre* nerves also displayed thinner myelin (S1 Fig).

In conclusion, our data indicate that loss of Kif13b specifically in Schwann cells affects longitudinal and radial myelin growth during development and remyelination after injury. Of note, myelination is not delayed in *Kif13b* mutant nerves at early stages of development, suggesting that Kif13b-mediated regulation occurs only during active myelination after P20.

### Kif13b Loss in Schwann Cells Is Associated with Increased Dlg1 Expression and Stability

**Fig 3:**
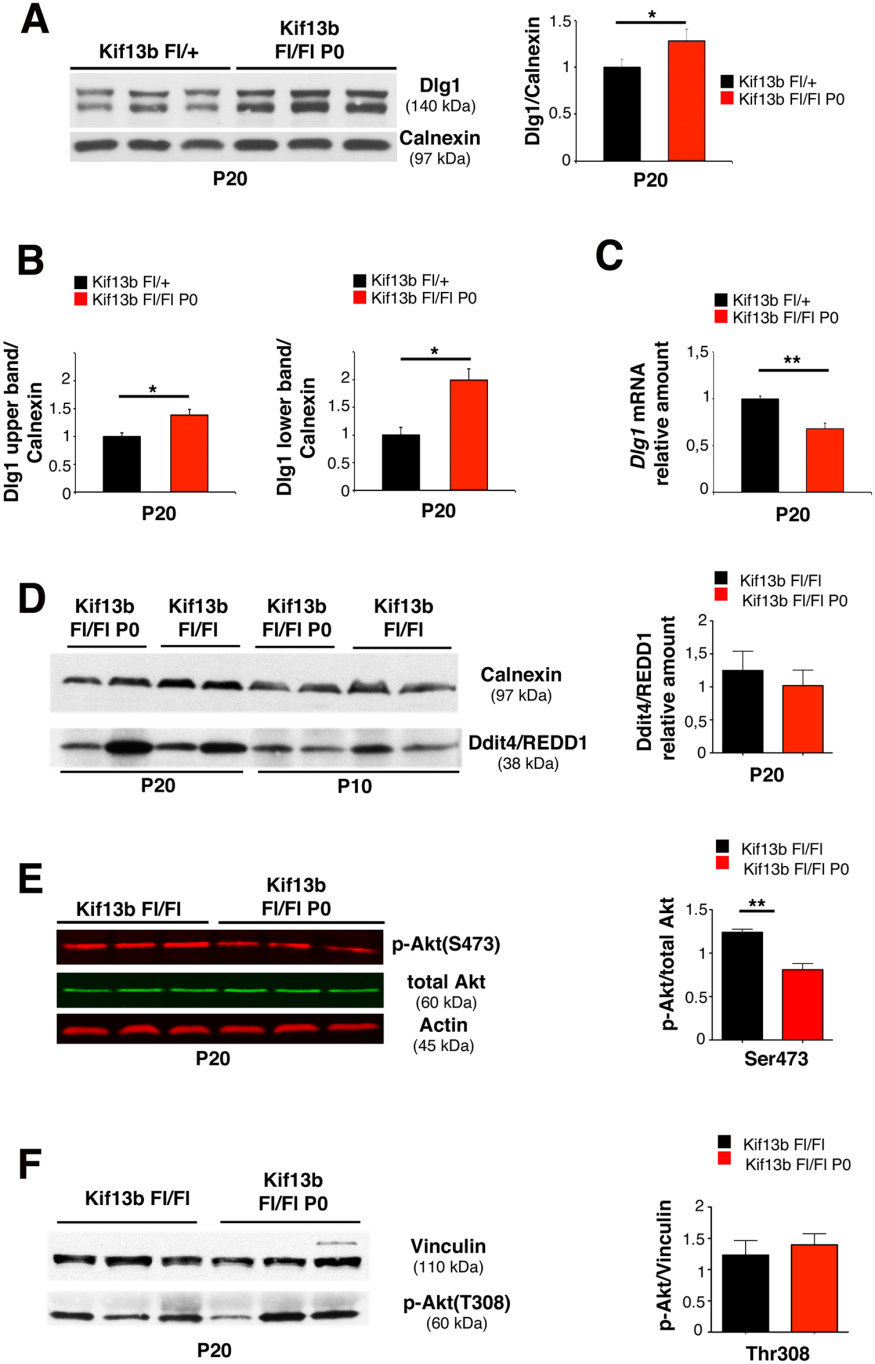
Increased Dlg1 expression in *Kif13b* ^*Fl/Fl*^ *P0-Cre* nerves. (A) Dlg1 expression is increased in *Kif13b* ^*Fl/Fl*^ *P0-Cre* nerve lysates, with quantification, *n* = 3, *p* = 0.042, respectively. (B) Quantification of the Dlg1 upper and lower isoform bands as shown in panel (A) *n* = 3, *p* = 0.041, and *p* = 0.022, three independent experiments. (C) qPCR analysis shows that *Dlg1* mRNA is decreased in *Kif13b* ^*Fl/Fl*^ *P0-Cre* nerves, *n* = 3, *p* = 0.008. (D) Expression levels of Ddit4/REDD1 in *Kif13b* ^*Fl/Fl*^ *P0-Cre* nerves at P10 and P20, with quantification at P20, *n* = 4, *p* = 0.564, representative of three independent experiments. (E) Phosphorylation of AKT at S473 is reduced in *Kif13b* ^*Fl/Fl*^ *P0-Cre* nerves, with quantification *n* = 3, *p* = 0.006, two independent experiments. (F) Phosphorylation of AKT at T308 in *Kif13b* ^*Fl/Fl*^ *P0-Cre* nerves, with quantification *n* = 3, *p* = 0.6, representative of three independent experiments.

To investigate the molecular basis of the observed myelin phenotype, we looked at the expression level of Dlg1, a known interactor of Kif13b and a negative regulator of Schwann cell myelination in vivo [7–9]. Interestingly, we found that Dlg1 expression level was increased in *Kif13b* ^*Fl/Fl*^ *P0-Cre* nerves at P20 (Fig 3A). Note that the increase is particularly evident in the lower band (Fig 3B), which corresponds to a hypo-phosphorylated isoform of Dlg1 [21,22]. In contrast, *Dlg1* mRNA levels were downregulated in mutant nerves (Fig 3C), which suggested that Dlg1 protein was more stable in the absence of Kif13b. To assess whether other negative regulators could contribute to the observed effect on myelination, we also looked at Ddit4/REDD1 expression levels. Ddit4/REDD1 is a known negative regulator of myelination, which downregulates the mechanistic target of rapamycin (mTOR) pathway by activating the tuberous sclerosis complex TSC1/2 [9]. We found that Ddit4 was similarly expressed between wild-type and mutant nerves at P10 and P20 (Fig 3D).

Dlg1 interacts with Kif13b in Schwann cells and is known to potentiate PTEN phosphatase activity on PIP_3_, thus downregulating AKT activation [7–9]. Consistent with this, phosphorylation of AKT at S473 was decreased in *Kif13b* ^*Fl/Fl*^ *P0-Cre* nerves as compared to controls at P20, when AKT phosphorylation starts to decline during postnatal nerve development (Fig 3E) [9]. On the contrary, in *Kif13b* ^*Fl/Fl*^ *P0-Cre* nerves, phosphorylation of AKT at T308 was not significantly different from controls (Fig 3F). This finding may indicate activation of the feedback loop involving mTOR and molecules upstream of PI3K, as also already observed in other mutants [9,23–26].

Finally, we found normal expression levels of NRG1 type III (and the phosphorylation of its receptor ErbB2), Krox20, and Oct6, known regulators of myelin initiation, further supporting that reduced myelin thickness of *Kif13b* ^*Fl/Fl*^ *P0-Cre* nerves is associated with enhanced negative regulation of postnatal myelination and not with a delay in myelin program initiation (S2 Fig).

**Fig 4:**
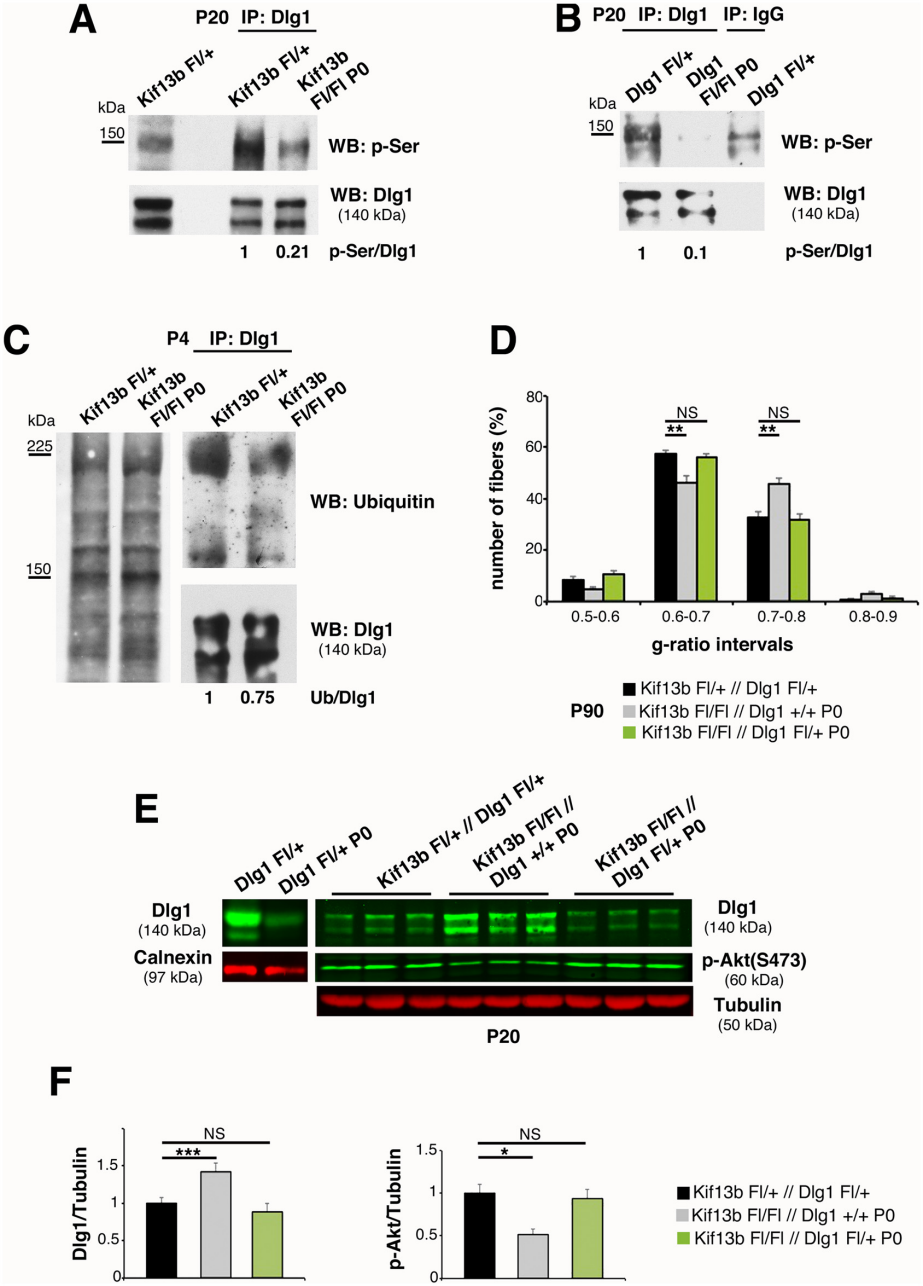
Kif13b negatively regulates Dlg1 expression and activity. (A) Immunoprecipitation of Dlg1 from *Kif13b* ^*Fl/Fl*^ *P0-Cre* nerves and controls at P20 followed by western blot analysis using an anti-phospho-serine antibody shows that Dlg1 is less phosphorylated in the mutant. Two independent experiments. (B) Immunoprecipitation of Dlg1 from *Dlg1* ^*Fl/Fl*^ *P0-Cre* nerves indicates that the band at 140–150 KDa is Dlg1. The residual Dlg1 protein in mutant nerves could result from non-recombined fibroblasts within the nerve as described in [9]. (C) Immunoprecipitation of Dlg1 at P4 followed by western blot analysis shows that Dlg1 is less ubiquitinated in mutant nerves. Two independent experiments. (D) G-ratio value distribution shows that haploinsufficiency of *Dlg1* in the *Kif13b* ^*Fl/Fl*^ *P0-Cre* background rescues the hypomyelination, *p* = 0.0093 and *p* = 0.0077, *n* = 4 animals per genotype, semithin section analysis. (E) Western blot analysis shows restored Dlg1 expression and AKT phosphorylation levels in *Kif13b* ^*Fl/Fl*^//*Dlg1* ^*Fl/+*^; *P0-Cre* nerve lysates, with quantification in (F), *p* = 0.0004 (Dlg1) and *p* = 0.020 (p-Akt), *n* = 3. The genetic reduction of 50% of Dlg1 is sufficient to reduce Dlg1 expression level in lysates from *Dlg1* ^*Fl/+*^ *P0-Cre*.

Phosphorylation is known to modulate protein–protein interactions necessary for the cytoskeletal localization of Dlg1 [27,28]. In particular, serine phosphorylation correlates with Dlg1 inactivation, and hyperphosphorylated Dlg1 interacts with ubiquitin ligases, which mediate its ubiquitination and degradation [8,21,22,27–30]. Thus, we hypothesized that increased Dlg1 protein levels in *Kif13b* ^*Fl/Fl*^ *P0-Cre* nerves could result from reduced serine phosphorylation and/or ubiquitination. By immunoprecipitating Dlg1 from sciatic nerve lysates at P20, we observed a decrease of Dlg1-serine phosphorylation in *Kif13b* ^*Fl/Fl*^ *P0-Cre* nerves compared to controls (Fig 4A). As expected, in *Dlg1* ^*Fl/Fl*^ *P0-Cre* sciatic nerve lysates, the phosphorylated band was not detected (Fig 4B). Then, to evaluate whether the decrease in serine-phosphorylation correlated with increased stability, we determined the pattern of Dlg1 ubiquitination. Consistent with our hypothesis, by immunoprecipitating Dlg1 from *Kif13b* ^*Fl/Fl*^ *P0-Cre* nerves at P4 and P10, we found that Dlg1 was less ubiquitinated in mutant nerve lysates when compared to controls (Fig 4C).

Our data suggest that the hypomyelination in *Kif13b* ^*Fl/Fl*^ *P0-Cre* nerves results from increased Dlg1 stability/activity and enhanced negative regulation of AKT. Hence, we hypothesized that 50% reduction of *Dlg1* gene expression in the *Kif13b* ^*Fl/Fl*^ *P0-Cre* background might rebalance Dlg1 levels and rescue the phenotype. Thus, we generated *Kif13b* ^*Fl/Fl*^//*Dlg1* ^*Fl/+*^; *P0-Cre* mice, and we compared these mutants with *Kif13b* ^*Fl/Fl*^//*Dlg1* ^*+/+*^; *P0-Cre* mouse nerves. By performing western blot analysis, we observed that Dlg1 expression and AKT phosphorylation levels in *Kif13b* ^*Fl/Fl*^//*Dlg1* ^*Fl/+*^; *P0-Cre* sciatic nerve lysates were rescued at a level similar to controls (Fig 4E and 4F). Accordingly, myelin thickness in *Kif13b* ^*Fl/Fl*^//*Dlg1* ^*Fl/+*^; *P0-Cre* nerves was also restored (Fig 4D). Overall, our data suggest that Kif13b negatively regulates Dlg1 stability and activity in Schwann cells. Thus, in *kif13b* ^*Fl/Fl*^ *P0-Cre* nerves, increased Dlg1 activity reduces AKT signaling and myelination.

### Kif13b Regulates Myelin Thickness in Oligodendrocytes

**Fig 5:**
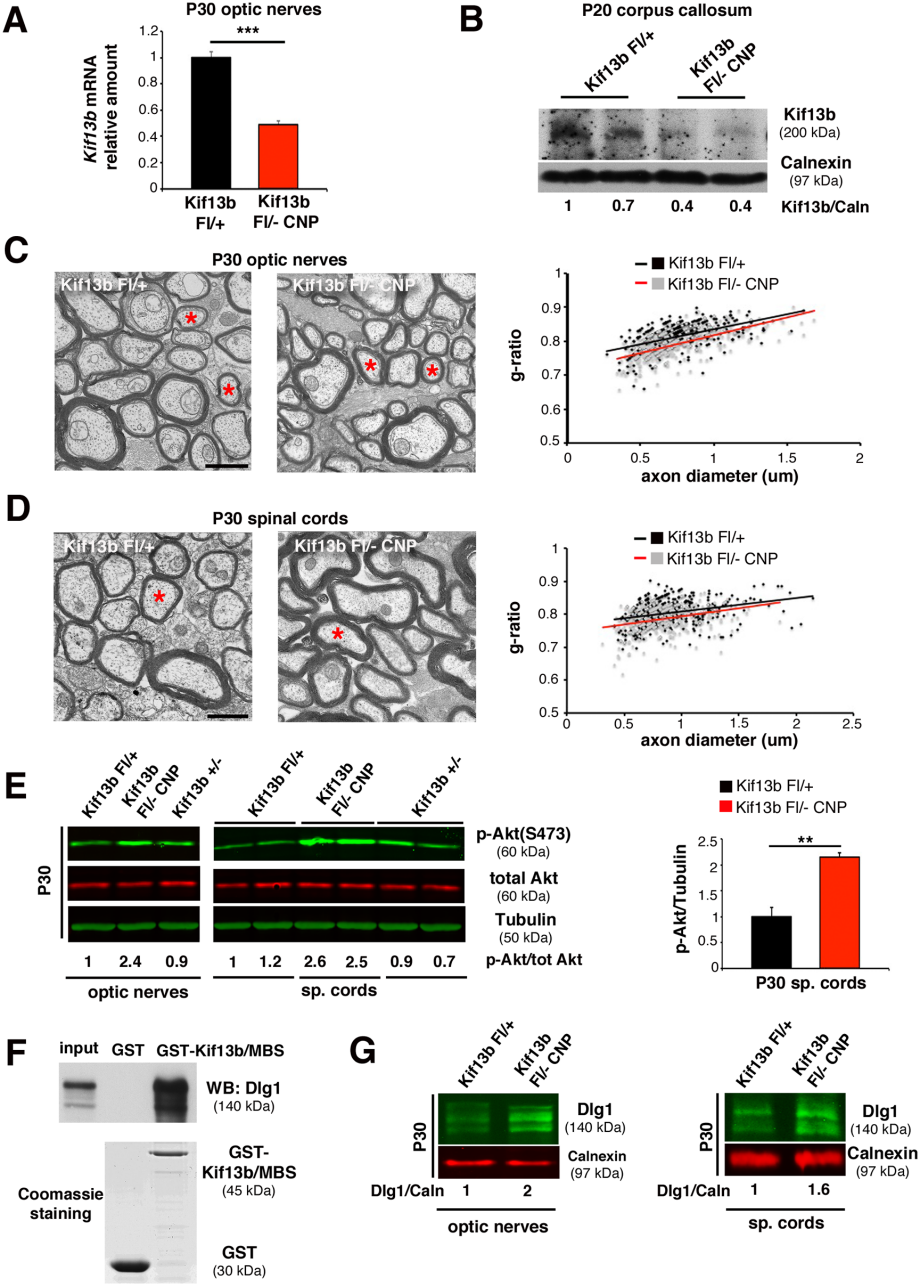
Kif13b regulates myelin thickness in oligodendrocytes. (A) RT-PCR analysis on optic nerves shows reduction of *Kif13b* mRNA in the mutant (*n* = 5, *p* = 0.0004). (B) Western blot analysis on lysates from corpus callosum at P20 shows reduction of Kif13b protein expression in the mutant. (C) Ultrathin analysis and quantification of the g-ratio as a function of axonal diameter in *Kif13b* ^*Fl/-*^ *CNP-Cre* and control optic nerves at P30. Red asterisks indicate fibers with similar diameter to be compared in the two genotypes (*Kif13b* ^*Fl/-*^ *CNP-Cre*, 0.792 ± 0.005, 296 fibers; *Kif13b* ^*Fl/+*^, 0.814 ± 0.006, 345 fibers, *n* = 4 animals per genotype, *p* = 0.030; *Kif13b* ^*+/-*^ 0.813 ± 0.004, 435 fibers, *n* = 5 animals per genotype). (D) Ultrathin analysis and quantification of the g-ratio as a function of axonal diameter in *Kif13b* ^*Fl/-*^ *CNP-Cre* and control spinal cords at P30 (*Kif13b* ^*Fl/-*^ *CNP-Cre*, 0.785 ± 0.003, 364 fibers; *Kif13b* ^*Fl/+*^, 0.803 ± 0.007, 403 fibers, *n* = 5 animals per genotype, *p* = 0.016). (E) Western blot analysis on lysates from optic nerves and spinal cords at P30 shows increased AKT phosphorylation at S473 in the mutant. Three independent experiments. Quantification of AKT phosphorylation levels from spinal cord lysates, *p* = 0.005, *n* = 3. (F) Pull down assay from P11 rat optic nerves using GST-Kif13b/MBS as a bait indicates Dlg1 and Kif13b interaction. Three independent experiments. (G) Western blot analysis on optic nerve and spinal cord lysates shows increased Dlg1 expression in *Kif13b* mutant oligodendrocytes. Three independent experiments. Bar in (C) and (D) is 1 μm.

Since Kif13b regulates PNS myelination, we sought to assess whether Kif13b has a similar role in the CNS. First, we confirmed *Kif13b* mRNA expression in optic nerves and in myelinated tracts of the corpus callosum (Fig 5A and 5B). Then, we generated a *Kif13b* ^*Fl/-*^ *CNP-Cre* mouse with conditional inactivation of *Kif13b* in newly generated oligodendrocytes [31]. To achieve maximum efficiency of *CNP-Cre* mediated recombination, we generated a compound heterozygous mouse for a *Kif13b* ^*Fl*^ allele and a *Kif13b* ^-^ (null) allele. We first assessed downregulation of *Kif13b* mRNA expression in *Kif13b* ^*Fl/-*^ *CNP-Cre* optic nerves by performing quantitative RT-PCR analysis (Fig 5A). Western blot analysis confirmed reduction of Kif13b protein expression in lysates from corpus callosum of *Kif13b* ^*Fl/-*^ *CNP-Cre* mice (Fig 5B). We then performed morphological analysis of optic nerves and spinal cords at P30. Surprisingly, we observed increased myelin thickness with decreased g-ratios in both *Kif13b* ^*Fl/-*^ *CNP-Cre* optic nerves and spinal cords as compared to either *Kif13b* ^*Fl/+*^ or *Kif13b* ^*-/+*^ controls (Fig 5C and 5D). However, at P90, myelin thickness in either *Kif13b* ^*Fl/-*^ *CNP-Cre* optic nerves or spinal cords was normal, suggesting a transient effect of Kif13b loss (S3 Fig).

At the molecular level, AKT phosphorylation at S473 was enhanced in both *Kif13b* ^*Fl/-*^ *CNP-Cre* optic nerves and spinal cords at P30 (Fig 5E), consistent with the observed hypermyelination and the role of AKT in promoting CNS myelination [32]. We then explored whether, as in the PNS, Kif13b regulates myelination by controlling Dlg1 expression levels. First, we assessed whether Kif13b interacts with Dlg1 in oligodendrocytes in vivo. By performing GST pull down assays from rat optic nerve lysates using GST-Kif13b/MBS as a bait, we identified Dlg1, suggesting the existence of a Kif13b/Dlg1 complex (Fig 5F). Interestingly, we noted that in spinal cord and optic nerve lysates Dlg1 isoforms were expressed in the range 140–150 KDa, as already observed in sciatic nerves (Fig 3A), where Dlg1 is not expressed in the axon [7,8]. This finding suggests that the Kif13b/Dlg1 interaction likely occurs in oligodendrocytes and not in axons/neurons, where the main Dlg1/SAP97 isoform runs at a different molecular weight (97KDa). Next, we evaluated Dlg1 protein expression in *Kif13b* ^*Fl/-*^ *CNP-Cre* mice and we found increased Dlg1 levels in both *Kif13b* ^*Fl/-*^ *CNP-Cre* optic nerves and spinal cords at P30 (Fig 5G). This result is consistent with the hypothesis that Kif13b negatively regulates Dlg1 expression also in the CNS.

Overall, our findings indicate that Kif13b is a transient negative regulator of myelination in the CNS as its downregulation in oligodendrocytes increases myelin thickness and enhances AKT activation. Moreover, we suggest that also in the CNS Kif13b interacts with Dlg1 and negatively regulates its stability.

### Dlg1 Enhances AKT Activation in Oligodendrocytes

**Fig 6:**
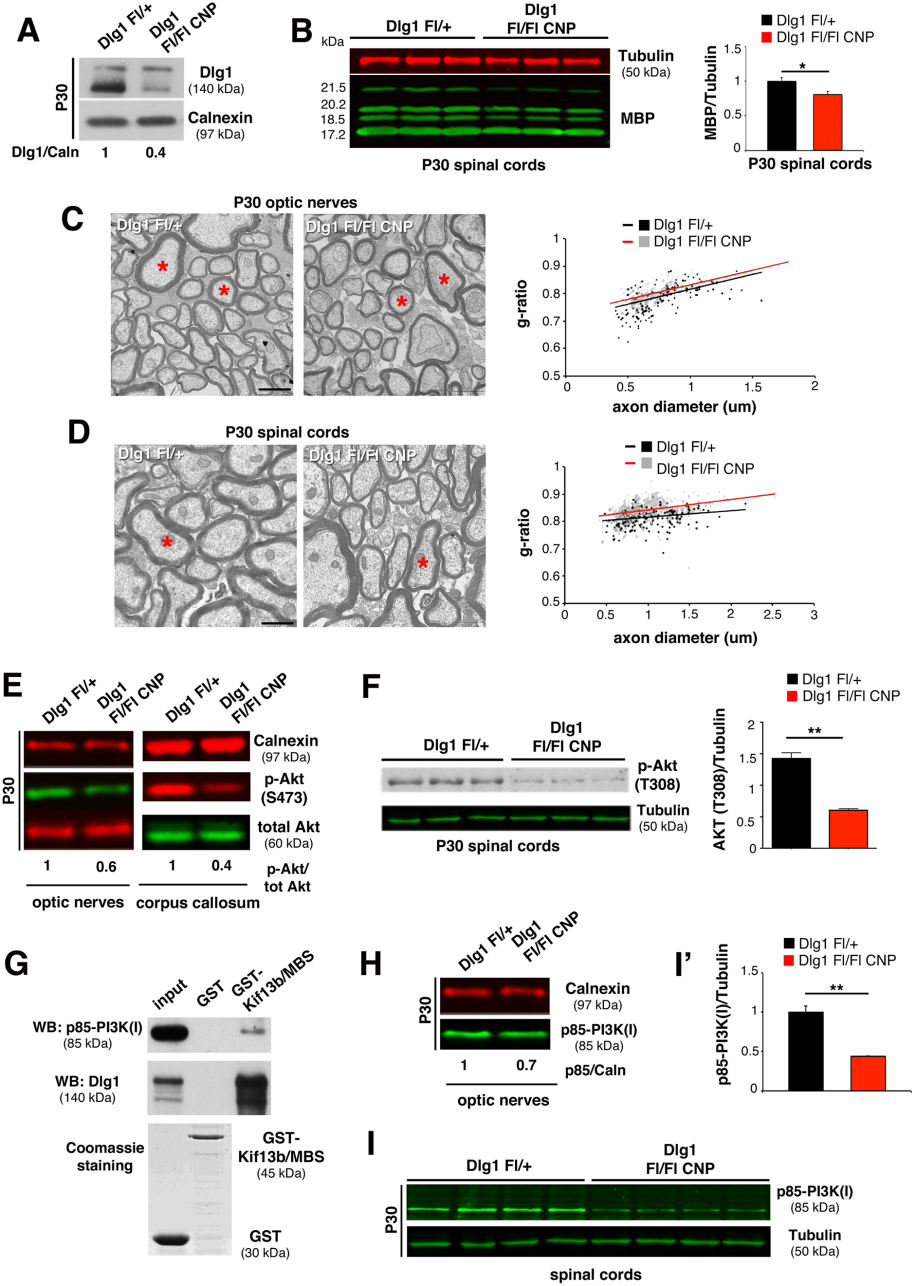
Dlg1 enhances AKT activation and regulates CNS myelination. (A) Dlg1 expression is reduced in *Dlg1* ^*Fl/Fl*^ *CNP-Cre* optic nerve lysates at P30. (B) Decreased MBP expression in mutant spinal cords, with quantification, *p* = 0.05, *n* = 3. (C) Ultrathin analysis and quantification of the g-ratio as a function of axonal diameter in optic nerves at P30 (*Dlg1* ^*Fl/Fl*^ *CNP-Cre*, 0.824 ± 0.006, 294 fibers; *Dlg1* ^*Fl/+*^ 0.804 ± 0.002, 310 fibers, *n* = 5 animals per genotype, *p* = 0.017). The red asterisks in the mutant indicate fibers with thinner myelin. (D) Ultrathin analysis and the g-ratio as a function of axonal diameter in spinal cords at P30 (*Dlg1* ^*Fl/Fl*^ *CNP-Cre*, 0.842 ± 0.004, 271 fibers, *Dlg1* ^*Fl/+*^, 0.815 ± 0.002, 213 fibers, *n* = 4 animals per genotype, *p* = 0.003). (E) Reduced S473 AKT phosphorylation in *Dlg1* ^*Fl/Fl*^ *CNP-Cre* mice, two independent experiments. (F) Phosphorylation of AKT at T308 is also reduced in *Dlg1* ^*Fl/Fl*^ *CNP-Cre* mice spinal cords, with quantification, *n* = 3, *p* = 0.001. (G) GST pull down assay from P11 rat optic nerves using GST-Kif13b/MBS as a bait indicates Dlg1 and p85 interaction. Two independent experiments. (H–I') p85 expression is reduced in lysates from *Dlg1* ^*Fl/Fl*^ *CNP-Cre* optic nerves and spinal cords (I), with quantification from spinal cord lysates, *p* = 0.003, *n* = 4 animals per genotype, five independent experiments. Bar in (C) and (D) is 1 μm.

In *Kif13b* ^*Fl/-*^ *CNP*-Cre mice, increased myelin thickness is associated with enhanced Dlg1 expression. However, if Dlg1 acts as a negative regulator of myelination in oligodendrocytes as well, we would have expected to observe hypomyelination and not hypermyelination. Thus, we hypothesized that in oligodendrocytes Dlg1 might have the opposite role in the control of myelination, being a promoter rather than an inhibitor. To test this hypothesis, we generated *Dlg1* ^*Fl/Fl*^ *CNP*-Cre conditional knockout mice in which *Dlg1* was ablated in oligodendrocytes. We first demonstrated a reduction of Dlg1 protein expression in *Dlg1* ^*Fl/Fl*^ *CNP-Cre* optic nerves at P30 (Fig 6A). Then, we performed morphological analyses of optic nerves and spinal cords starting at P30. Consistent with our hypothesis, mutant optic nerves and spinal cords displayed reduced myelin thickness and increased g-ratios (Fig 6C and 6D). Hypomyelination was also evidenced by decreased myelin basic protein (MBP) expression levels in spinal cord lysates from *Dlg1* ^*Fl/Fl*^ *CNP-Cre* mice (Fig 6B). As in the case of *Kif13b* ^*Fl/-*^ *CNP-Cre* mutants, myelin thickness of *Dlg1* ^*Fl/Fl*^ *CNP-Cre* optic nerves and spinal cords was normal at P90, suggesting a transient role of Dlg1 in the control of myelination (S3 Fig).

To investigate the mechanism by which Dlg1 promotes myelination in oligodendrocytes, we examined the phosphorylation state of AKT in lysates from optic nerves and corpus callosum of *Dlg1* ^*Fl/Fl*^ *CNP-Cre* mutants. We found that AKT phosphorylation at both S473 and T308 was reduced in both *Dlg1* ^*Fl/Fl*^ *CNP-Cre* optic nerves and corpus callosum as compared to controls, consistent with the decreased myelination (Fig 6E and 6F).

Since (1) AKT phosphorylation depends on PIP_3_ levels and on the activity of the PI3K class I and (2) Dlg1 has been described to interact with the regulatory subunit of PI3K class I, p85, in epithelial cells [29], we hypothesized that also in oligodendrocytes Dlg1 may interact with p85, influencing PI3K activity upstream of AKT.

To address this point, we first explored p85 expression levels in optic nerves and spinal cords at P30 and found that p85 protein levels were reduced in *Dlg1* ^*Fl/Fl*^ *CNP-Cre* mice (Fig 6H–6I'). Next, GST pull down experiments from P11 rat optic nerve lysates demonstrated that Dlg1 and p85 are interactors of GST-Kif13b/MBS (Fig 6G), thus providing evidence for the existence of a complex involving Kif13b, Dlg1, and p85. Interestingly, by performing co-immunoprecipitation and pull down experiments, we did not observe interaction between p85 and the Kif13b/Dlg1 complex in the PNS in sciatic nerves. Consistent with this, p85 was similarly expressed in *Kif13b* ^*Fl/Fl*^ *P0-Cre* and *Dlg1* ^*Fl/Fl*^ *P0-Cre* mutant sciatic nerves as compared to controls (S4 Fig). These findings suggest that in the PNS, in contrast to the CNS, the Kif13b/Dlg1 complex does not involve p85.

In conclusion, similarly to Schwann cells, downregulation of Kif13b expression in oligodendrocytes is associated with increased Dlg1 levels. However, in the CNS, Dlg1 promotes myelination. Thus, downregulation of Kif13b expression in oligodendrocytes causes hypermyelination.

### p38γ MAPK Regulates Dlg1 Stability in Both PNS and CNS

**Fig 7:**
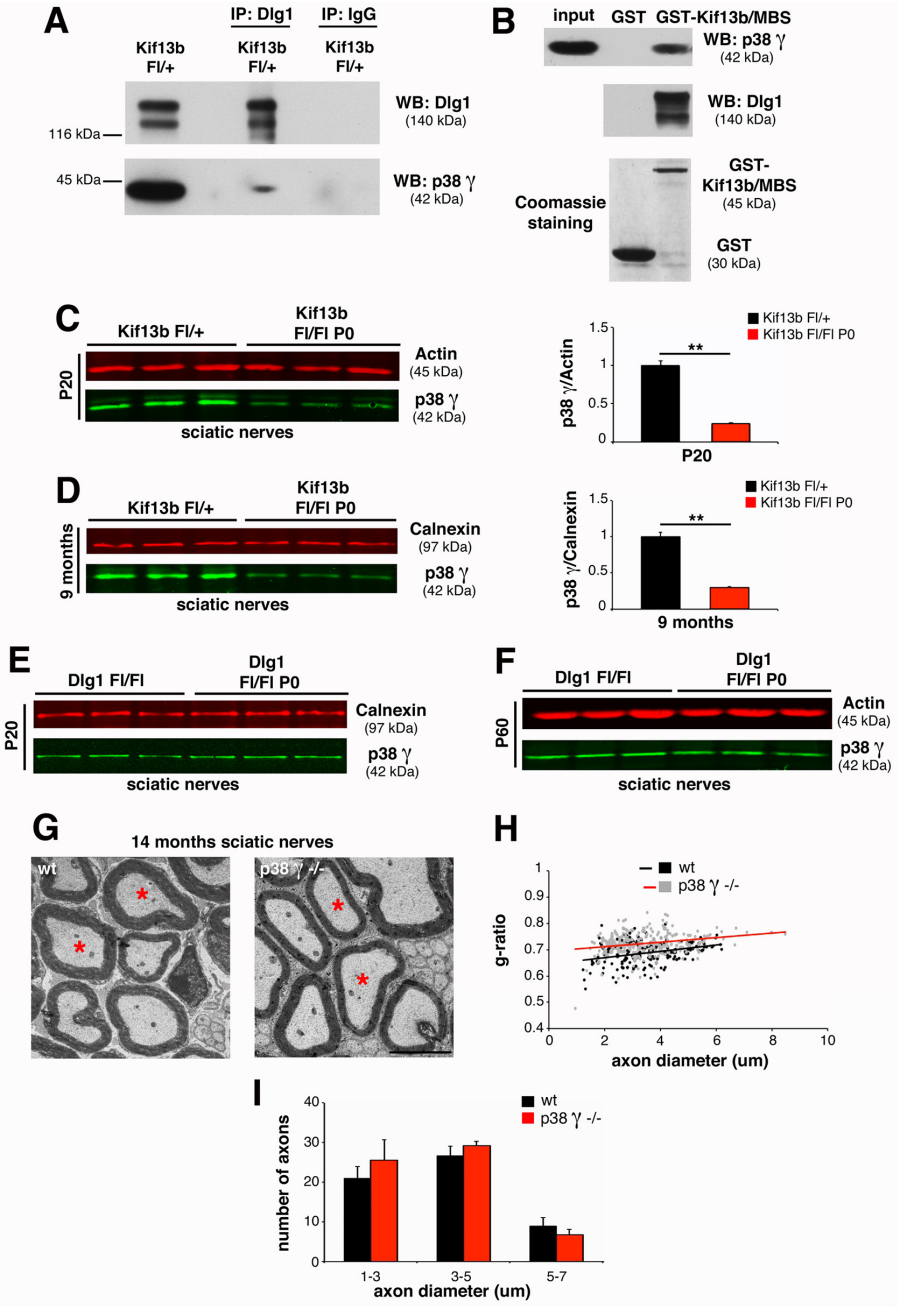
p38γ MAPK acts in complex with Kif13b and Dlg1 in the PNS. (A) Dlg1 and p38γ co-immunoprecipitate from mouse sciatic nerve lysates at P20. Two independent experiments. (B) Kif13b/MBS-GST used to pull down Dlg1 and p38γ from rat sciatic nerve lysates at P11. Two independent experiments. (C) p38γ expression levels are reduced in *Kif13b* ^*Fl/Fl*^ *P0-Cre* nerve lysates at both P20 and (D) at 9 mo, with quantification, *p* = 0.005 at P20 and *p* = 0.007 at 9 mo, *n* = 3. (E, F) p38γ expression is not reduced in lysates from *Dlg1* ^*Fl/Fl*^ *P0-Cre* nerves at P20 and P60. (G, H) G-ratio as a function of axonal diameter in *p38γ*-null sciatic nerves at 14 mo, performed by ultrastructural analysis (wild-type, 0.688 ± 0.008, 170 fibers, *n* = 3 animals, *p38γ*-null 0.726 ± 0.009, 249 fibers, *n* = 4 animals, *p* = 0.023). Total number of axons does not change in the two genotypes. (I) Fiber diameter distribution in *p38γ*-null nerves at 14 mo of age. Bar in (G) is 2.5 μm.

Finally, we asked how downregulation of Kif13b expression results in increased Dlg1 stability in both PNS and CNS. In previous yeast two-hybrid screening analyses, we had found that the PDZ2+3 domain of Dlg1 directly interacts with the p38γ MAPK isoform [7,33], as also previously reported for HEK293 cells [28]. Since p38γ can phosphorylate and negatively regulate the interaction of Dlg1 with cytoskeletal protein partners, we further investigated the interaction of Kif13b, p38γ, and Dlg1 in the nerve in vivo. We first confirmed Dlg1 and p38γ interaction by performing co-immunoprecipitation experiments from sciatic nerve lysates (Fig 7A). Next, we observed that Kif13b/MBS-GST was able to pull down both Dlg1 and p38γ from nerve lysates, suggesting that Kif13b, p38γ, and Dlg1 may be part of the same complex (Fig 7B). To provide further evidence for this hypothesis, we investigated p38γ expression levels in mutants with conditional ablation of either *Kif13b* or *Dlg1* in Schwann cells. Interestingly, p38γ expression levels were decreased in *Kif13b* ^*Fl/Fl*^ *P0-Cre* sciatic nerves at both P20 and 9 mo (Fig 7C and 7D) but not in *Dlg1* ^*Fl/Fl*^ *P0-Cre* nerves (Fig 7E and 7F), suggesting that p38γ acts downstream of Kif13b and upstream of Dlg1. To confirm these results, we analyzed the sciatic nerves of p38γ knock-out mutants. As expected, nerves from p38γ-null mice were hypomyelinated (Fig 7G–7I), supporting the hypothesis that p38γ is a novel promoter of Schwann cell myelination.

**Fig 8:**
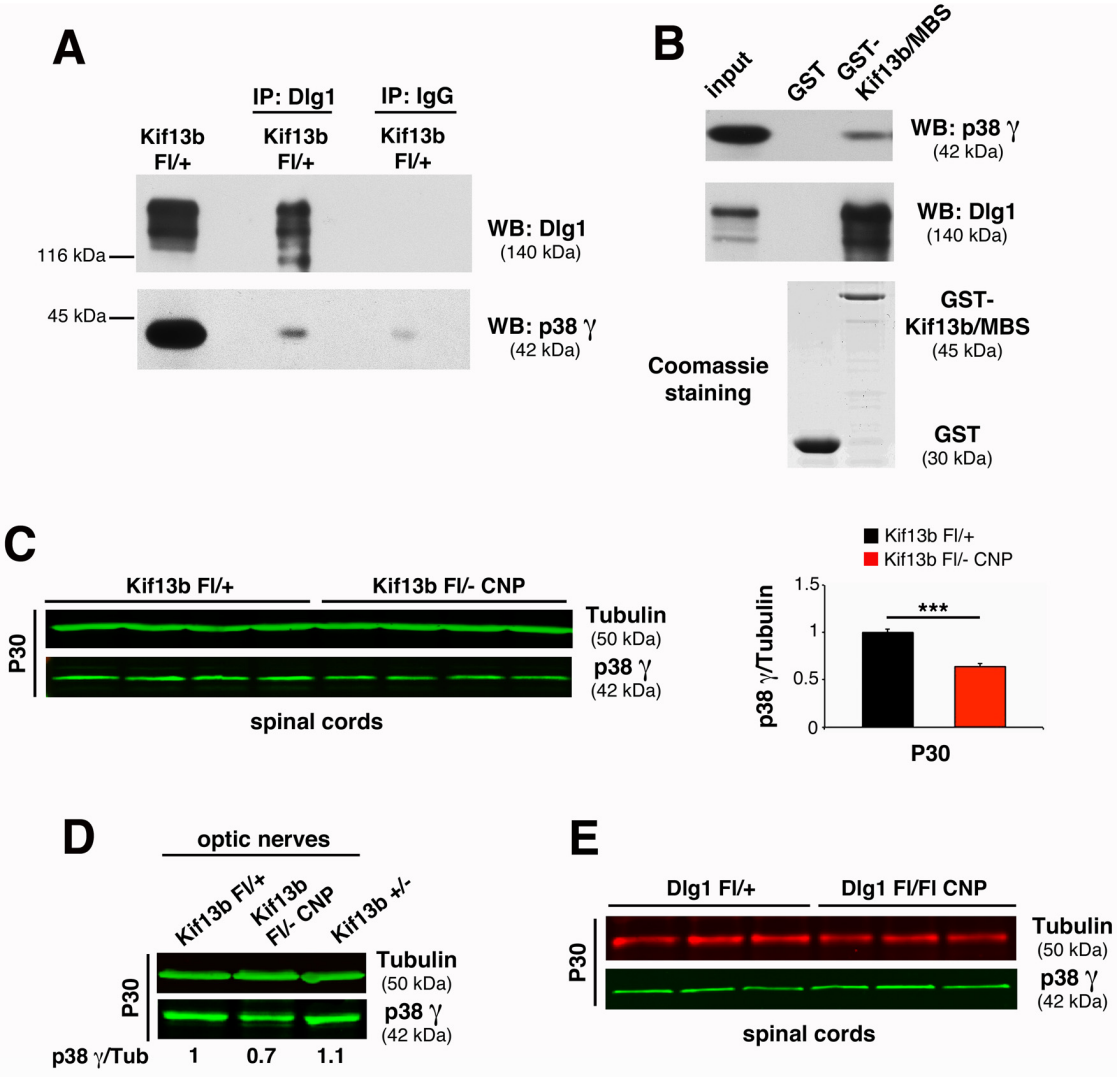
p38γ MAPK acts in complex with Kif13b and Dlg1 in the CNS. (A) Dlg1 and p38γ co-immunoprecipitate from rat optic nerve lysates at P11. Two independent experiments. (B) Kif13b/MBS-GST pulls down Dlg1 and p38γ from rat optic nerve lysates at P11. Two independent experiments. (C,D) p38γ expression levels are reduced in *Kif13b* ^*Fl/-*^ *CNP-Cre* optic nerve and spinal cord lysates at P30, with quantification, *p* = 0.0005, *n* = 4 animals per genotype, representative of four independent experiments. (E) p38γ expression is not reduced in *Dlg1* ^*Fl/Fl*^ *CNP-Cre* spinal cords at P30.

Finally, since our data suggest that Kif13b may similarly regulate Dlg1 also in the CNS, we assessed whether a Kif13b, p38γ, and Dlg1 complex could be detected in oligodendrocytes. As expected, Dlg1 and p38γ co-immunoprecipitate from optic nerve lysates (Fig 8A) and Kif13b/MBS-GST is able to pull down both Dlg1 and p38γ (Fig 8B). Even if not as striking as in Schwann cells, p38γ expression levels were decreased in *Kif13b* ^*Fl/-*^ *CNP-Cre* optic nerve lysates but not in *Dlg1* ^*Fl/Fl*^ *CNP-Cre*, suggesting that p38γ acts downstream of Kif13b and upstream of Dlg1 (Fig 8C–8E).

As p38α is the MAPK isoform known to regulate myelination in both PNS and CNS [34–41], we assessed whether Kif13b/Dlg1 may also form a complex with p38α. Interestingly, by performing pull down experiments, we found that Dlg1 does not interact with p38α in either optic or sciatic nerve lysates. Consistent with this, expression levels of p38α in either sciatic nerves or spinal cords of *Kif13b* conditional knock-out mutants were similar to controls (S5 Fig).

Overall, these findings suggest a similar mechanism of Kif13b and p38γ-mediated regulation of Dlg1 in both PNS and CNS, with opposite outcomes on the control of myelination, as Dlg1 is a brake on myelination in the PNS and a positive regulator in the CNS.

## Discussion

**Fig 9:**
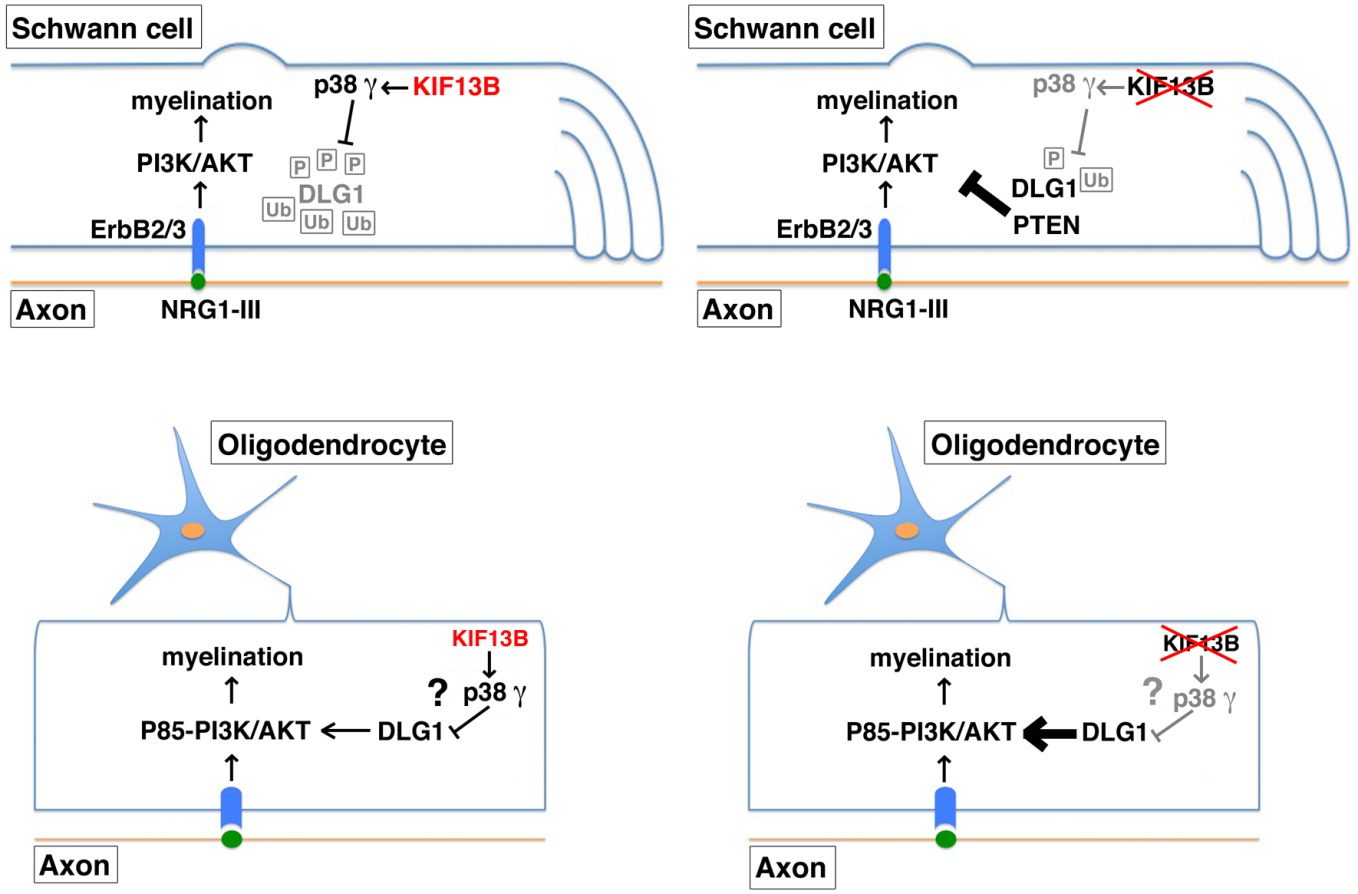
Kif13b regulates myelination in PNS and CNS through the Dlg1 scaffold. The PI3K-AKT-mTOR signaling axis is one of the signaling pathways regulating myelination in both PNS and CNS, as recently reviewed [49–56,64]. Our results suggest that in Schwann cells, Kif13b is a positive regulator of myelination. Kif13b promotes p38γ MAPK-mediated Dlg1 phosphorylation and ubiquitination. Dlg1, in complex with PTEN, is known to reduce AKT activation and thus negatively regulates myelination. Dlg1 loss is associated with increased myelin thickness and myelin outfoldings, as a result of increased PIP_3_ and AKT phosphorylation levels [8,9,47]. On the contrary, in oligodendrocytes, loss of Kif13b-mediated negative regulation of Dlg1 and the consequent increase in Dlg1 levels are associated with transient hypermyelination. Of note, we found that in the CNS Dlg1 is a promoter and not an inhibitor of myelination, and it likely modulates PI3K class I activity.

Microtubule-based kinesin motors have many cellular functions, including the transport of a variety of cargos to different parts of the cell [42]. Motors can also be used to place cargos on a long distance, such as signaling complexes or developmental determinants in neurons or embryos, respectively. However, unconventional functions have recently emerged, and kinesins have also been reported to act as scaffolding proteins and signaling molecules [43]. In particular, Kif13b has been recently shown in hepatocytes to work as a scaffold and to enhance caveolin-1 dependent internalization of LRP11 receptor [12]. In T cells, Kif13b acts as a signaling molecule that controls CARD11 scaffold localization at the synapse and downregulates TCR signaling [13]. In this work, we further extend these findings on unconventional roles of kinesins and propose a novel mechanism by which the Kif13b motor protein regulates Dlg1 scaffold activity and titrates the PI3K/AKT signaling with two opposite outcomes in PNS and CNS myelination (Fig 9).

### Kif13b Regulates PNS Myelination through the Dlg1 Scaffold

Here we report that downregulation of Kif13b expression in Schwann cells is associated with reduced myelin thickness, decreased AKT activation, and increased levels of Dlg1, a known brake on PNS myelination acting on the PIP_3_-AKT-mTOR pathway [8,9]. As Kif13b and Dlg1 interact in Schwann cells [7], we hypothesized that Kif13b may control myelination through the Dlg1 scaffold itself, by regulating its stability and function. Indeed, in support of our hypothesis, in *Kif13b* ^*Fl/Fl*^ *//Dlg1* ^*Fl/+*^; *P0-Cre* double mutant sciatic nerves, Dlg1 expression levels and myelin thickness are similar to wild type.

Interestingly, we report here that in *Kif13b* ^*Fl/Fl*^ *P0-Cre* nerves, in which Dlg1 expression levels are increased, myelination is not delayed at very early stages of postnatal nerve development, and reduced myelin thickness is evident when AKT activation starts to physiologically decline, after P20 [8,9]. This observation is consistent with the phenotype of mutant mice lacking Dlg1, specifically in Schwann cells [9]. We previously reported a transient increase in myelin thickness and occasional myelin outfoldings in *Dlg1* ^*Fl/Fl*^ *P0-Cre* nerves starting from P10 [9]. However, even if enhanced, myelination was not accelerated in *Dlg1* ^*Fl/Fl*^ *P0-Cre* nerves, and, at very early stages of postnatal nerve development, the number of myelinated fibers and myelin thickness were similar to control nerves. Thus, Dlg1 may act as a brake on myelination to downregulate AKT activation at the peak of myelination, when AKT phosphorylation starts to decline. In support to this hypothesis, myelin outfoldings, a focal form of hypermyelination that is thought to be linked to AKT overactivation and loss of Dlg1-mediated negative control on myelination, are observed in the nerve after 3 w of postnatal development [44].

Dlg1 stability is controlled by phosphorylation and ubiquitination [8,21,22,27,28,30,45,46]. In *Drosophila*, the PAR1 kinase directly phosphorylates Dlg1 at conserved sites and negatively regulates its mobility and targeting at postsynaptic membranes of neuromuscular junctions [27]. Osmotic stress-induced serine phosphorylation of Dlg1 by p38γ MAP kinase can induce Dlg1 dissociation from the glucokinase-associated dual specificity phosphatase (GKAP) and the cytoskeleton, negatively regulating Dlg1 [28]. Finally, phosphorylated DLG1 interacts with the β-TrCP ubiquitin ligase receptor, which mediates ubiquitination of the protein [30]. Thus, we investigated whether enhanced Dlg1 protein expression levels in *Kif13b* ^*Fl/Fl*^ *P0-Cre* nerves correlated with a decrease in serine phosphorylation and/or ubiquitination. Consistent with our hypothesis, we found that in *Kif13b* ^*Fl/Fl*^ *P0-Cre* nerves Dlg1 is less phosphorylated and less ubiquitinated, suggesting that Kif13b promotes radial myelin growth by directly or indirectly influencing Dlg1 stability and expression.

We also suggest that p38γ MAPK could be the kinase that, downstream of Kif13b, phosphorylates Dlg1 to regulate its activity. Indeed, p38γ MAPK is known to interact with and to phosphorylate serine residues of Dlg1 in other cells [28]. We identified p38γ in a yeast two-hybrid screening analysis using a nerve cDNA library and Dlg1 as a bait [7,33]. Moreover, we show that p38γ, Dlg1, and Kif13b form a complex in the nerve. More importantly, sciatic nerves of *p38*γ-null mice are hypomyelinated, thus confirming the hypothesis that p38γ, by phosphorylating and negatively regulating Dlg1, acts as a promoter of myelination downstream of Kif13b. Unfortunately, antibodies that can specifically recognise phosphorylated p38γ are not available to assess whether activated p38γ could interact with Kif13b and Dlg1.

Interestingly, the role of p38γ MAPK in the regulation of PNS myelination has not yet been assessed. Previous studies suggested that p38 MAPK mediates laminin signaling in vitro to promote Schwann cell elongation and alignment at the very first stages of differentiation [34]. Hossain et al., suggested that p38 directs Schwann cell differentiation by regulating Krox-20 expression, thus further supporting the role of p38 MAPK as a positive regulator of PNS myelination [35]. However, on the basis of the MAPK inhibitors used, the observed effect was likely to be mediated by the p38α or p38β [35]. A more recent study reported that *in vitro* p38 MAPK promotes the de-differentiated state of Schwann cells during Wallerian degeneration, by inducing c-Jun expression and by inhibiting myelin gene expression, and also suggested that p38 MAPK is a negative regulator of Schwann cell differentiation and myelination during development [36]. On the basis of the antibodies used recognizing the phosphorylated state of MAPK as well as the MAPK inhibitor used (SB203580), other isoforms rather than p38γ are more likely to mediate this function [36].

How can both Kif13b and p38γ control Dlg1 phosphorylation, ubiquitination, and stability? Kif13b could transport and localize the kinase at membranes where Dlg1 is enriched to downregulate, in complex with PTEN, PIP_3_ levels, and AKT activation [47]. Indeed, in *Kif13b*-null but not in *Dlg1*-null nerves p38γ expression levels are reduced, thus suggesting that p38γ is downstream of Kif13b and upstream of Dlg1. Alternatively, the binding of Kif13b with Dlg1, which is mediated by the membrane-associated guanylate kinase homologue binding stalk (MBS) and guanylate kinase homologue (GUK) domains, respectively, may relieve intramolecular inhibition in either Kif13b or Dlg1, as already reported [48]. For example, following Kif13b binding, a conformational change in Dlg1 (open state) can be induced so that target residues for serine phosphorylation can be exposed and accessible to p38γ kinase-mediated phosphorylation. Unfortunately, p38γ-specific inhibitors are not available to further investigate these mechanisms.

Our data convey a novel function for Kif13b/p38γ as negative regulators of Dlg1 in the PI3K/AKT signalling pathway. Interestingly, Kif13b has already been proposed as a negative regulator in other studies. For example, in PC12 cells, KIF13B negatively regulates centaurin-α_1_/PIP_3_BP (PIP_3_ binding protein), a GAP for Arf6, thus promoting Arf6 GTPase plasma membrane activation [16]. Further, in T cells, KIF13B negatively regulates TCR signaling to NF-kB, by redistributing the CARD11 scaffold from the center of the synapse to a more distal region [13].

### Kif13b Regulates CNS Myelination through the Dlg1 Scaffold

We also show that Kif13b is a negative regulator of CNS myelination. Indeed, we observed that downregulation of Kif13b expression in oligodendrocytes results in increased myelin thickness and AKT activation, consistently with the role of AKT in promoting CNS myelination [32]. Similar to PNS, we found that Kif13b interacts with Dlg1 and that loss of Kif13b is associated with increased Dlg1 levels, thus suggesting a negative regulation mediated by Kif13b on Dlg1. Given these similarities, we investigated whether the increased Dlg1 level and stability in oligodendrocytes could also result from a decrease in p38γ-mediated phosphorylation. Indeed, we found that Kif13b, Dlg1, and p38γ MAPK interact in optic nerves and that p38γ expression is decreased in Kif13b but not in Dlg1 mutants, as already observed in the PNS. These findings suggest that p38γ may act downstream of Kif13b and upstream of Dlg1 to negatively regulate Dlg1 activity.

The role of the p38γ isoform in the regulation of CNS myelination has not been yet assessed. As for PNS, only p38α has been investigated in the CNS. Inhibition of p38α activity or expression in vitro in a co-culture system has been reported to prevent oligodendrocyte progenitor differentiation and myelination [37–39]. Another study suggested that p38α MAPK supports myelin gene expression in the brain through several mechanisms acting on both positive and negative regulators of differentiation [40]. More recently, myelination was found to be impaired in mice with conditional inactivation of p38α MAPK in oligodendrocyte progenitor cells [41]. Interestingly, the same authors observed an opposite effect of p38α MAPK in remyelination, as mutant mice exhibited a more efficient remyelination as compared to controls following demyelination [41]. These studies further support the notion that the regulation of myelination is a very complicated process, in which different signals arising from the extracellular matrix, axons, and astrocytes in the CNS must be correctly integrated in time and space within the same cell to achieve homeostasis [49–56].

If Dlg1 is a brake on myelination in the CNS as in the PNS, how can loss of Kif13b and elevation of Dlg1 result in increased CNS myelin thickness? Surprisingly, our data indicate that in oligodendrocytes Dlg1 is a positive and not a negative regulator of myelination, as its loss is associated with reduced myelin thickness and AKT activation. Interestingly, in addition to Dlg1, other molecules have been found to control myelination with opposite roles in PNS and CNS [57–60]. For example, myosin light chain II phosphorylation promotes myelination in the PNS and inhibits myelination in the CNS [57].

To determine the mechanism by which Dlg1 could promote CNS myelination acting on the PI3K-AKT pathway, we sought to investigate the regulatory subunit of PI3K class I, p85, a known interactor of Dlg1 in epithelial cells [29]. Consistent with this, we found that Dlg1 interacts with p85 in the optic nerve, likely to modulate PI3K class I activity, PIP_3_ levels, and ultimately AKT activation. Interestingly, phosphorylation of DLG1 on serine and threonine is known to prevent DLG1 interaction with SH2 domains of p85/PI3K [29]. Thus, we could speculate that Dlg1, when hypophosphorylated, may display a higher affinity for the SH2 domains of p85, whose activation is necessary for PI3K activity regulation [61].

Whether in oligodendrocytes Dlg1 also promotes myelination by other mechanisms, which can converge on AKT activation, remains to be determined.

## Material and Methods

### Antibodies

The following primary antibodies were used: mouse anti-KIF13B (provided by Dr. A. Chishti); mouse anti-Dlg1 (Stressgen); mouse anti-phosphoserine (Alexis Biochemicals); rabbit anti-ubiquitin (Santa Cruz Biotechnology); rabbit anti-DRP2 (provided by Dr. D. Sherman); rabbit anti-phospho-Akt (Ser473) (Cell Signaling); rabbit anti-phospho-Akt (Thr308) (Cell Signaling); rabbit anti-Akt (pan) (Cell Signaling); rabbit anti-phospho-p44/42 MAPK (Erk1/2) (Thr202/Tyr204) (Cell Signaling); rabbit anti-p44/42 MAP Kinase (Cell Signaling); rabbit anti-Neuregulin-1α/β1/2 (C20) (Santa Cruz Biotechnology); rabbit anti-p-Neu (Tyr 1248)-R (i.e., p-ErbB-2) (Santa Cruz Biotechnology); rabbit anti-Neu (C-18) (i.e., ErbB-2) (Santa Cruz Biotechnology); rabbit anti-PI3 Kinase p85 (Cell Signaling); rat anti-MBP (Millipore); rabbit anti-p38α (Santa Cruz); rabbit anti-p38γ (R&D Systems); rabbit anti-calnexin (Sigma-Aldrich); mouse anti-β-tubulin (Sigma-Aldrich); rabbit anti-actin (Sigma-Aldrich). For immunofluorescence, secondary antibodies included fluorescein (FITC)-conjugated and rhodamine (TRITC)-conjugated donkey anti-mouse or rabbit IgG (Jackson ImmunoResearch). For western blotting, secondary antibodies included horseradish peroxidase (HRP)-conjugated goat anti-rabbit and rabbit anti-mouse immunoglobulins (Dako), and IRDye 800- and 680-conjugated goat anti-mouse, goat anti-rabbit, and goat anti-rat IgG (Li-Cor Biosciences). As negative control in immunoprecipitation experiments, ChromPure mouse IgG whole molecules were used (Jackson ImmunoResearch).

### Animals

All experiments involving animals were performed in accordance with Italian national regulations and covered by experimental protocols reviewed by local Institutional Animal Care and Use Committees.

The pFlrt-1 vector, including *lox-P* sites, *FRT*-flanked neomycin resistance gene (neo), and *PGK-TK*, was used to target the *Kif13b* gene. The selected *Kif13b* mouse genomic regions to be inserted in the targeting vector were amplified from a BAC clone spanning the *Kif13b* gene and obtained from The Center for Applied Genomics (The Hospital for Sick Children, Ontario, Canada).

To generate the targeting vector for homologous recombination, a 503 bp *Bam*HI fragment including exon 6 and flanking intronic regions was first inserted between *lox-P* sites in pFlrt-1. In a second step, a 4,606 bp fragment containing exon 7 was inserted into the *BstB*I site downstream of the *PGK-neo* cassette to constitute the long arm for homologous recombination. Finally, a fragment of 2,000 bp containing exon 5 was cloned into the *Sal*I site upstream to the first *lox-P* and represented the short arm for homologous recombination.

After electroporation of TBV2 embyonic stem cells (129S2/SvPas), recombinant clones were screened by Southern blot analysis. Digestion with *Kpn*I and hybridization with two probes designed on exon 6 (inside the recombination) and upstream of exon 5 (outside the 5′ end of the recombination) revealed two bands of 7,671 bp (wild type) and of 9,671 bp (containing the *neo* cassette).

Similarly, *Sma*I digestion of genomic DNA and hybridization using a probe designed at the 3′ end of the targeted region, outside the recombination boundaries, detected two bands of 7,694 bp (the targeted allele, since one *Sma*I restriction site is present within the *neo* cassette) and of 9,151 bp (the wild-type allele).

Two different correctly targeted clones were injected into C57BL6 blastocysts (Core Facility for Conditional Mutagenesis San Raffaele/Telethon Transgenic Service) to obtain transmission of the Floxed allele through the germline.

The *neo* cassette was removed in vivo by crossing heterozygous *Kif13b* ^*Fl (neo)/+*^ with *Flpe* transgenic mice. Heterozygous *Kif13b* ^*Fl/+*^ animals were crossed with *P0-Cre* [17,18] transgenic mice to excise exon 6 specifically in Schwann cells. To generate *Kif13b* ^*Fl/Fl*^ *P0-Cre* conditional knockout mice, *Kif13b* ^*Fl/+*^ *P0-Cre* animals were crossed with homozygous *Kif13b* ^*Fl/Fl*^. *Kif13b* ^*Fl/Fl*^ mouse nerves had normal myelin thickness and mean g-ratio values similar to wild-type mice, thus suggesting that *Kif13b* ^*Fl/+*^ does not represent a hypomorphic allele.

To obtain *Kif13b* ^*Fl/-*^ *CNP-Cre* [31] mice with conditional inactivation of *Kif13b* in oligodendrocytes, *Kif13b* ^*Fl/+*^ mice were first crossed with *CMV-Cre* transgenic mice. Then, after germline segregation of the *CMV-Cre* transgene, *Kif13b* ^*-/+*^ (without *CMV-Cre*) were crossed with *Kif13b* ^*Fl/+*^ *CNP-Cre* mice to obtain *Kif13b* ^*Fl/-*^ *CNP-Cre* conditional null. In this way, we increased *CNP-Cre* mediated recombination efficiency on the Floxed allele in the *Kif13b* ^*Fl/-*^ *CNP-Cre* genotype.

The *Dlg1* ^*Fl*^ (C57/BL6 strain) allele has been already reported (Zhou et al., 2008). To generate *Dlg1* conditional knockout mice in oligodendrocytes, homozygous *Dlg1* ^*Fl/Fl*^ mice were crossed with heterozygous *Dlg1* ^*Fl/+*^ mice carrying the *CNP-Cre* transgene.

To obtain 50% reduction of *Dlg1* specifically in Schwann cells in a *Kif13b* ^*Fl/Fl*^ *P0-Cre* background, *Kif13b* ^*Fl/Fl*^ *P0-Cre* mice were first crossed with *Dlg1* ^*Fl/Fl*^ *P0-Cre* mice. Then, *Kif13b* ^*Fl/+*^//*Dlg1* ^*Fl/+*^; *P0-Cre* double heterozygous mice were crossed to obtain *Kif13b* ^*Fl/Fl*^//*Dlg1* ^*Fl/+*^; *P0-Cre* mice. These latter were compared with *Kif13b* ^*Fl/Fl*^//*Dlg1* ^*+/+*^; *P0-Cre* mice and controls (only floxed alleles without Cre) within the same litters.

The generation of p38γ-null mice has been already reported [28].

For all the experiments involving animals, *n* ≥ 5 animals per genotype of either sex were analysed.

### PCR Analysis

Genotype analysis on *Kif13b* mutant mice was carried out on tail genomic DNA using primer pairs A plus B (415 bp floxed band and 342 bp wild type band) or A plus C (966 bp floxed band, 834 bp wild type band, and 378 bp recombined band). Genotype analysis of the Dlg1 floxed allele and of the p38γ-null locus has already been reported [9,28].

RT-PCR was performed as described previously [7,9]. Designed probes were used to amplify mouse *Kif13b* and the endogenous reference transcript *calnexin*. The comparative Ct method was used. As calibrator, a control sample ΔCt was chosen for each selected transcript. The ΔΔCt (ΔCt of each normalized selected transcript minus ΔCt of the calibrator) was calculated. Expression levels of *Kif13b* mRNA are indicated as 2^-ΔΔCt^ values. For statistical analysis, SD was calculated for triplicate samples of each reaction and SEM is indicated on the average of the determinations from different animals.

### Morphological Analysis

Three to five animals per genotype for each time point were analysed. Semithin analysis of quadriceps and sciatic nerves and ultrastructural analysis of optic nerves and spinal cords were performed as described previously [62].

To perform morphometric analysis, digitalized images of fiber cross sections were obtained from corresponding levels of the quadriceps or sciatic nerves with a 100x objective and Leica DFC300F digital camera (Milan, Italy). Five images per animal were analysed with the Leica QWin software (Leica Microsystem) and the g-ratio calculated as the ratio between the mean diameter of an axon (without myelin) and the mean diameter of the same axon including the myelin sheath.

For morphometric analysis on ultrastructural sections, 20 images per animal were taken at 4000x (LEO 912AB Transmission Electron Microscope, Milan, Italy) and the g-ratio values determined by measuring axon and fiber diameters.

Internodal lengths were measured as described using Openlab (PerkinElmer) [19], and 100 internodes of two quadriceps nerves were evaluated for each animal (*n* = 3).

### Sciatic Nerve Crush

Adult mice were anesthetized with avertin (trichloroethanol, 0.02 ml/g of body weight), and crush injury was performed as previously described [63]. After skin incision, the sciatic nerve was exposed and crushed distal to the sciatic notch for 20 s with fine forceps previously cooled in dry ice. To identify the site of injury, forceps were previously dropped into vital carbon. The nerve was replaced under the muscle and the incision sutured.

### Preparation of Tissue Lysates

Protein lysates from mouse sciatic nerves, corpus callosum, optic nerves, and spinal cords for western blot analysis were prepared using a lysis buffer containing 2% SDS, 50 mM Tris buffer pH 8.0, 150 mM NaCl, 10 mM NaF, 1 mM NaVO_3_, and complete protease and phosphatase inhibitors (Roche). For the detection of phosphorylated antigens, samples were lysed with a buffer containing 1%TX-100. Protein quantification was performed using BCA assay (Pierce, Thermo Scientific).

### Co-Immunoprecipitation

Mouse and rat sciatic nerves were lysed in a buffer containing 1% NP-40, 150 mM NaCl, 50 mM Tris buffer pH 8.0, 10 mM NaF, 1 mM NaVO_3_, and complete protease and phosphatase inhibitors (Roche). Following centrifugation at 13,000 rpm for 15 min at 4°C, equal amounts of protein lysates were incubated with 6–8 ug of mouse anti-Dlg1 antibody (Stressgen) or mouse IgG for control (Jackson ImmunoResearch). After 3 h of incubation with the antibody at 4°C, 35 μl of protein G agarose (settled) (Sigma-Aldrich) was added to immunocomplexes within the lysates and incubated for 1 h and 30 min at 4°C. The agarose beads were washed two times with cold PBS-Tween 0.1% and once with cold PBS. The immunoprecipitated product was denatured in Laemmly buffer (Biorad) with β-mercaptoethanol and resolved by SDS-PAGE.

### Glutathione S-transferase- (GST) Binding Assays

Kif13b/MBS cDNA was cloned into pGEX-4T2 expression vector and expressed together with GST alone in *Escherichia coli* BL21(DE3) cells [7]. Recombinant proteins were purified directly from bacterial extract on glutathione-Sepharose 4 Fast Flow beads.

Rat sciatic and optic nerves were lysed in a buffer containing 1% NP-40, 50 mM Tris buffer pH 7.4, 10% glycerol, 100 mM NaCl, 10 mM NaF, and 1 mM NaVO_3_. Equal amounts of protein lysates were incubated for 4 h at 4°C with immobilized GST-Kif13b/MBS proteins and GST as control. After three washes with a buffer containing 0.5% NP-40, 50 mM Tris buffer pH 7.4, 10% glycerol, 100 mM NaCl, 10 mM NaF, and 1 mM NaVO_3_, the bead pellets were dissolved in Laemmly buffer with β-mercaptoethanol, resolved by SDS-PAGE, and analyzed by immunoblotting. To show the relative amount used of GST-Kif13B/MBS and GST, beads were dissolved again in Laemmly buffer with β-mercaptoethanol, resolved by SDS-PAGE, and the gels stained with Coomassie.

### Western Blotting

SDS-PAGE gels were transferred to PVDF membranes (Millipore) or to nitrocellulose (Millipore) at 4°C in 20% methanol blotting buffer. Filters were blocked in 5% dry milk in PBS-0.1% Tween 20 overnight at 4°C and immunoblotted with primary antibodies diluted in 3% dry milk in PBS-0.1% Tween. For phosphorylated antigens, an additional blocking was performed for 30 min at RT in 3% bovine serum albumin (BSA) (Sigma-Aldrich), 0.5% gelatin, 0.1% Tween, 1 mM EDTA pH 8.0, 0.15 M NaCl, 10 mM Tris buffer pH 7.5, followed by incubation with primary antibodies diluted in the same blocking solution. Secondary antibodies, either horseradish peroxidase-conjugated (Dako) or IRDye 800- and 680-conjugated (Li-Cor Biosciences), were used and immunoblots revealed by using either ECL/ECL-prime developing systems and films for chemiluminescent detection (Amersham) or by Odyssey CLx Infrared Imaging System (Li-Cor Biosciences).

### Statistical Analysis

Statistical analysis was performed using the Student *t* test, two tails, unequal variants, and α = 0.005 were considered. All results are shown as mean ± SEM.

### Images

Figures were prepared using Adobe Photoshop version 11.0 (Adobe Systems).

## Supporting Information

S1 Data Excel file containing in separate sheets the numerical data for Figs 2B, 2D, 3A–3F, 4D–4F, 5A, 5C–5E, 6B–6D, 6F, 6H, 6I, 7C, 7D, 7H, 7I, 8C, S1A, S1B, S2A–S2E, S3A–S3D and S4C–S4E. (XLSX)

S1 Fig Kif13b promotes remyelination following crush nerve injury. (A) Nerve regeneration 1 mo after crush injury shows that *Kif13b* ^*Fl/Fl*^ *P0-Cre* nerves regenerate properly, as assessed by semithin section analysis. Only the number of big caliber axons was reduced in crushed *Kif13b* ^*Fl/Fl*^ *P0-Cre* nerves (as a percentage, *Kif13b* ^*Floxed/+*^, 3.404 ± 0.865; *Kif13b* ^*Fl/Fl*^ *P0-Cre*, 0.883 ± 0.352, *n* = 5 animals per genotype, *p* = 0.027). (B) Quantification of the g-ratio as a function of the axonal diameter in crushed sciatic nerves shows that *Kif13b* ^*Fl/Fl*^ *P0-Cre* nerves are hypomyelinated 2 mo after injury (*Kif13b* ^*Fl/Fl*^ *P0-Cre*, 0.713 ± 0.009, 1924 fibers; *Kif13b* ^*Fl/+*^, 0.681 ± 0.007,1944 fibers, *n* = 3 animals per genotype, *p* = 0.005).
(TIF)

S2 Fig Expression levels of known promoters of myelination. (A) Expression levels with quantification of Nrg1-III, of ErbB2 (B), and of Erk phosphorylation (C) in *Kif13b* ^*Fl/Fl*^ *P0-Cre* sciatic nerves. (D) Expression levels with quantification of Krox20 and Oct6 (E) in *Kif13b* ^*Fl/Fl*^ *P0-Cre* sciatic nerves.
(TIF)

S3 Fig Loss of either Kif13b or Dlg1 in oligodendrocytes transiently impairs myelination. (A,B) The g-ratio as a function of axonal diameter in *Kif13b* ^*Fl/-*^ *CNP-Cre* optic nerves and spinal cords shows normal myelin thickness at P90 (optic nerve: *Kif13b* ^*Fl/-*^ *CNP-Cre*, 0.792 ± 0.007, 454 fibers; *Kif13b* ^*Fl/+*^, 0.811 ± 0.006, 408 fibers, *n* = 5 animals per genotype, *p* = 0.077. Spinal cord: *Kif13b* ^*Fl/-*^ *CNP-Cre*, 0.787 ± 0.014, 228 fibers; *Kif13b* ^*Fl/+*^, 0.789 ± 0.013, 218 fibers, *n* = 3 animals per genotype, *p* = 0.897). (C,D) The g-ratio as a function of axonal diameter in *Dlg1* ^*Fl/Fl*^ *CNP-Cre* optic nerves and spinal cords shows normal myelin thickness at P90 (optic nerve: *Dlg1* ^*Fl/Fl*^ *CNP-Cre*, 0.811 ± 0.008, 696 fibers; *Dlg1* ^*Fl/+*^, 0.815 ± 0.004, 734 fibers, *n* = 4 animals per genotype, *p* = 0.647. Spinal cord: *Dlg1* ^*Fl/Fl*^ *CNP-Cre*, 0.792 ± 0.013, 403 fibers; *Dlg1* ^*Fl/+*^, 0.789 ± 0.002, 390 fibers, *n* = 3 animals per genotype, *p* = 0.855).
(TIF)

S4 Fig p85 and Dlg1 do not interact in sciatic nerves. Immunoprecipitation of Dlg1 from mouse sciatic nerves at P20 followed by western blot analysis using an anti-p85 antibody shows that Dlg1 does not interact with p85 in Schwann cells. (B) GST pull down assay from P11 rat optic nerves using GST-Kif13b/MBS as a bait indicates that Dlg1 does not interact with p85 in Schwann cells, two independent experiments. (C) Expression levels of p85 in sciatic nerves of *Kif13b* ^*Fl/Fl*^ *P0-Cre* mutants and controls at P20, with quantification. (D) Expression levels of p85 in sciatic nerves of *Dlg1* ^*Fl/Fl*^ *P0-Cre* mutants and controls at P20, with quantification. (E) Expression levels of p85 in sciatic nerves of both *Kif13b* ^*Fl/Fl*^ *P0-Cre* and *Dlg1* ^*Fl/Fl*^ *P0-Cre* mutants at 5 mo, with quantification.
(TIF)

S5 Fig Kif13b/Dlg1 complex does not involve p38α MAPK. (A) GST pull down assay from P11 rat optic nerves performed using GST-Kif13b/MBS as a bait indicates that Dlg1 does not interact with p38α in rat optic nerves at P11 or (B) in sciatic nerves at P11. (C) Expression levels of p38α in both *Kif13b* ^*Fl/Fl*^ *P0-Cre* sciatic nerves and *Kif13b* ^*Fl/Fl*^ *CNP-Cre* spinal cords is similar to controls.
(TIF)

## Abbreviations

AKT: v-AKT murine thymoma viral oncogene homolog
Arf6: ADP-ribosylation factor
CNS: central nervous system
Dlg1: Discs large 1
DRG: dorsal root ganglia
GAKIN: guanylate kinase-associated kinesin
GAP: GTPase activating protein
GKAP: glucokinase-associated dual specificity phosphatase
GUK: guanylate kinase homologue
MAPK: mitogen-activated protein kinase
MBP: myelin basic protein
MBS: membrane-associated guanylate kinase homologue binding stalk
MPZ: the myelin protein zero gene
mTOR: mechanistic target of rapamycin
PIP_3_: phosphatidylinositol-3,4,5-trisphosphate
PI3K: phosphatidylinositol 3-kinase
PNS: peripheral nervous system
PTEN: phosphatase and tensin homolog
