# Supplementary figures and images for: Kif13b Regulates PNS and CNS Myelination through the Dlg1 Scaffold
Source: PLoS Biol. 2016 Apr 12;14(4):e1002440. doi: 10.1371/journal.pbio.1002440 (PMC4829179; doi:10.1371/journal.pbio.1002440)

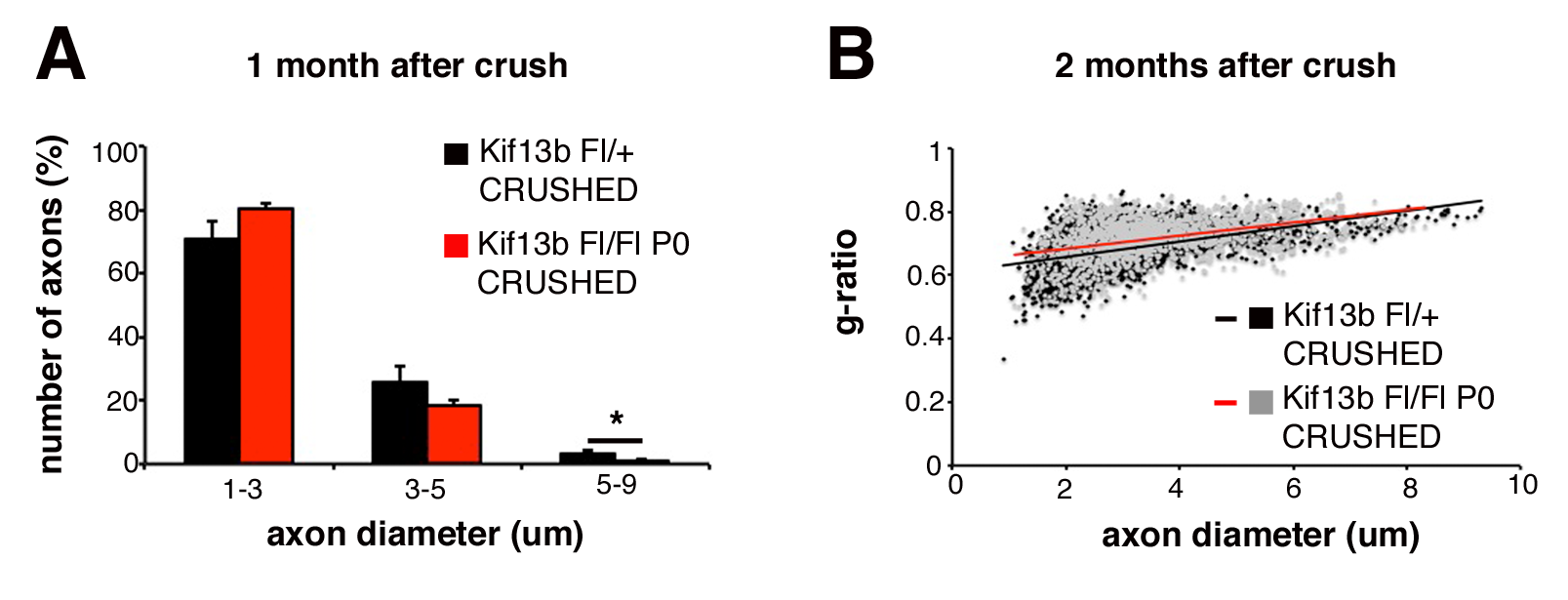

Supplement: S1 Fig — (A) Nerve regeneration 1 mo after crush injury shows that Kif13b Fl/Fl P0-Cre nerves regenerate properly, as assessed by semithin section analysis. Only the number of big caliber axons was reduced in crushed Kif13b Fl/Fl P0-Cre nerves (as a percentage, Kif13b Floxed/+, 3.404 ± 0.865; Kif13b Fl/Fl P0-Cre, 0.883 ± 0.352, n = 5 animals per genotype, p = 0.027). (B) Quantification of the g-ratio as a function of the axonal diameter in crushed sciatic nerves shows that Kif13b Fl/Fl P0-Cre nerves are hypomyelinated 2 mo after injury (Kif13b Fl/Fl P0-Cre, 0.713 ± 0.009, 1924 fibers; Kif13b Fl/+, 0.681 ± 0.007,1944 fibers, n = 3 animals per genotype, p = 0.005). (TIF) [file pbio.1002440.s002.tif]

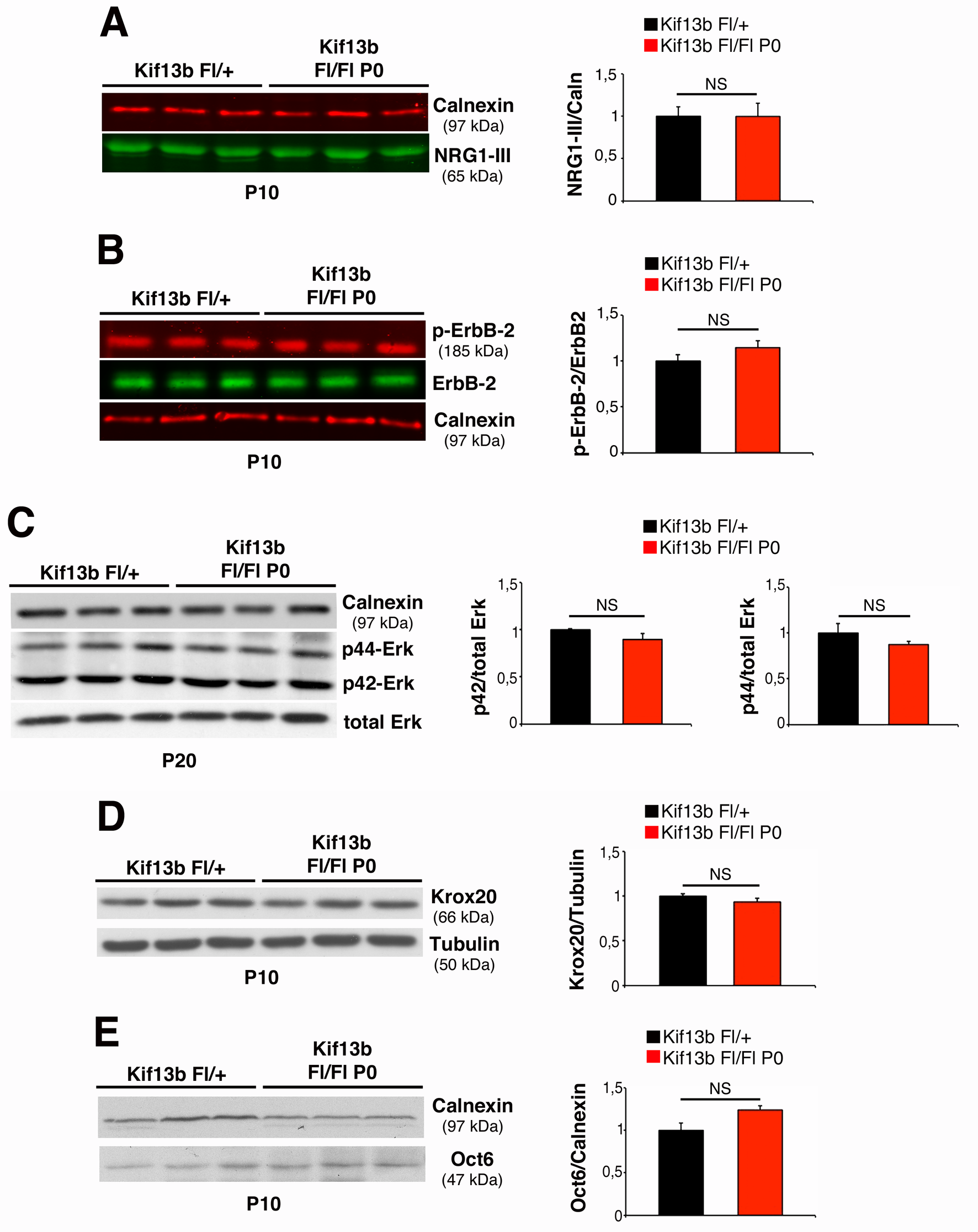

Supplement: S2 Fig — (A) Expression levels with quantification of Nrg1-III, of ErbB2 (B), and of Erk phosphorylation (C) in Kif13b Fl/Fl P0-Cre sciatic nerves. (D) Expression levels with quantification of Krox20 and Oct6 (E) in Kif13b Fl/Fl P0-Cre sciatic nerves. (TIF) [file pbio.1002440.s003.tif]

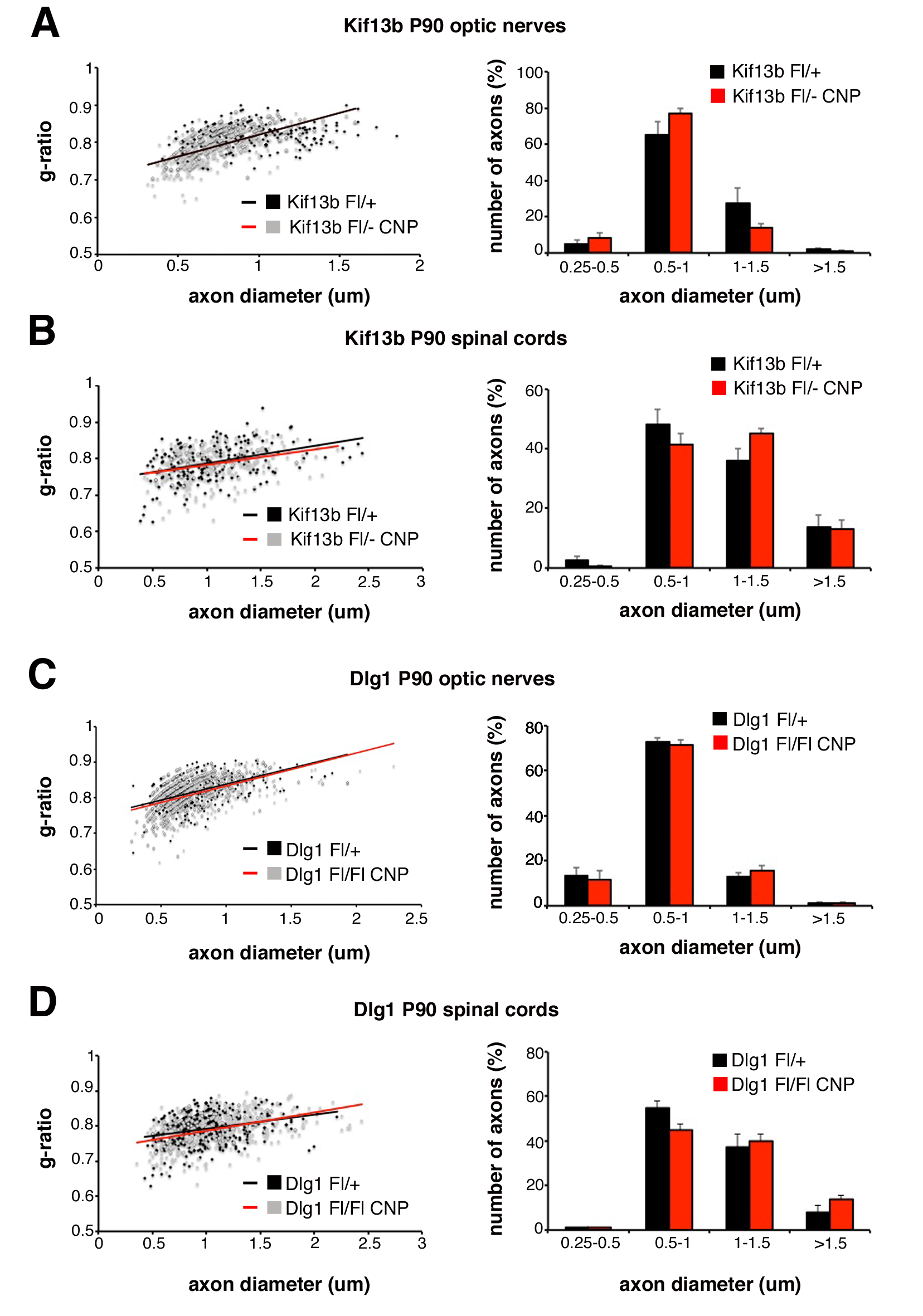

Supplement: S3 Fig — (A,B) The g-ratio as a function of axonal diameter in Kif13b Fl/- CNP-Cre optic nerves and spinal cords shows normal myelin thickness at P90 (optic nerve: Kif13b Fl/- CNP-Cre, 0.792 ± 0.007, 454 fibers; Kif13b Fl/+, 0.811 ± 0.006, 408 fibers, n = 5 animals per genotype, p = 0.077. Spinal cord: Kif13b Fl/- CNP-Cre, 0.787 ± 0.014, 228 fibers; Kif13b Fl/+, 0.789 ± 0.013, 218 fibers, n = 3 animals per genotype, p = 0.897). (C,D) The g-ratio as a function of axonal diameter in Dlg1 Fl/Fl CNP-Cre optic nerves and spinal cords shows normal myelin thickness at P90 (optic nerve: Dlg1 Fl/Fl CNP-Cre, 0.811 ± 0.008, 696 fibers; Dlg1 Fl/+, 0.815 ± 0.004, 734 fibers, n = 4 animals per genotype, p = 0.647. Spinal cord: Dlg1 Fl/Fl CNP-Cre, 0.792 ± 0.013, 403 fibers; Dlg1 Fl/+, 0.789 ± 0.002, 390 fibers, n = 3 animals per genotype, p = 0.855). (TIF) [file pbio.1002440.s004.tif]

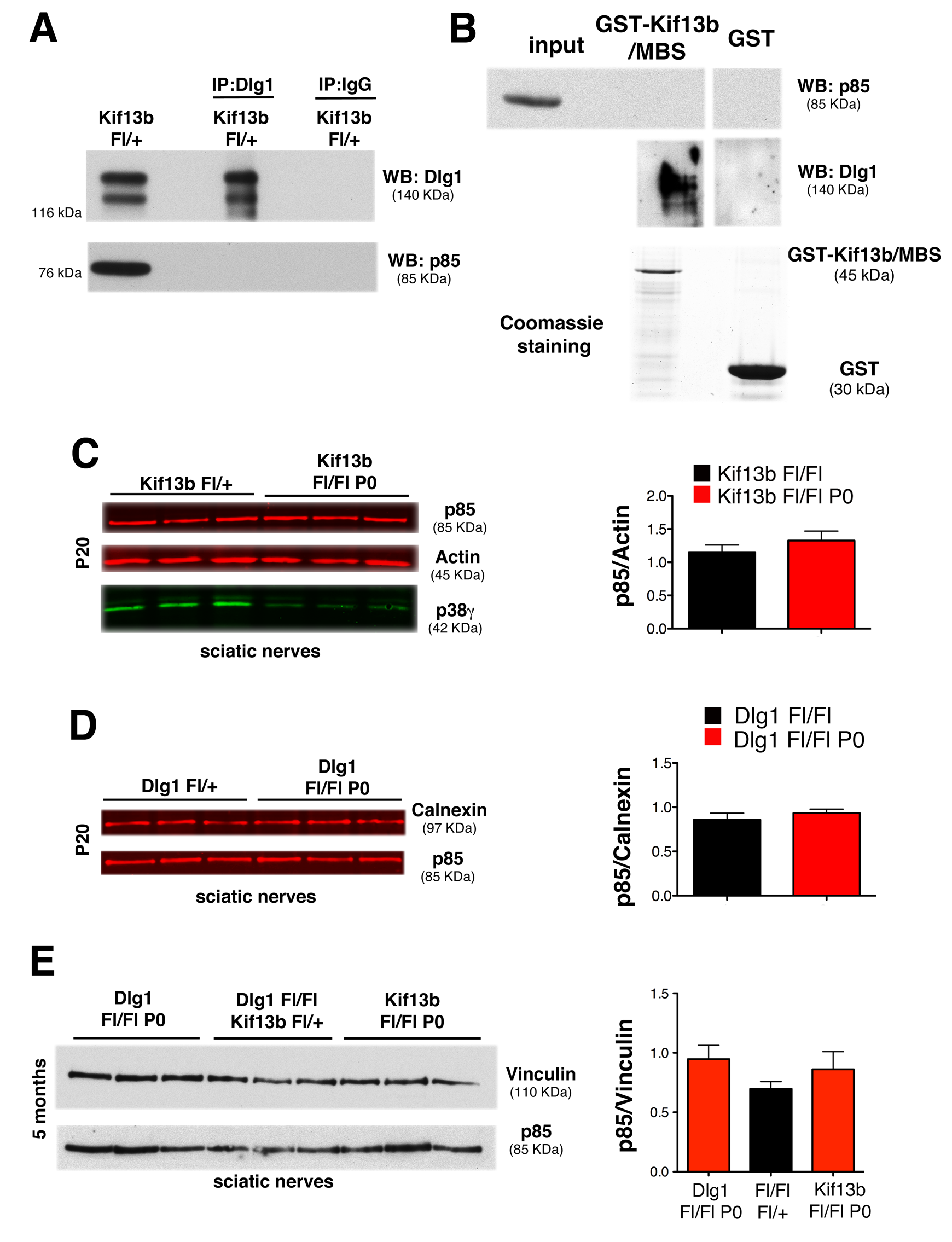

Supplement: S4 Fig — Immunoprecipitation of Dlg1 from mouse sciatic nerves at P20 followed by western blot analysis using an anti-p85 antibody shows that Dlg1 does not interact with p85 in Schwann cells. (B) GST pull down assay from P11 rat optic nerves using GST-Kif13b/MBS as a bait indicates that Dlg1 does not interact with p85 in Schwann cells, two independent experiments. (C) Expression levels of p85 in sciatic nerves of Kif13b Fl/Fl P0-Cre mutants and controls at P20, with quantification. (D) Expression levels of p85 in sciatic nerves of Dlg1 Fl/Fl P0-Cre mutants and controls at P20, with quantification. (E) Expression levels of p85 in sciatic nerves of both Kif13b Fl/Fl P0-Cre and Dlg1 Fl/Fl P0-Cre mutants at 5 mo, with quantification. (TIF) [file pbio.1002440.s005.tif]

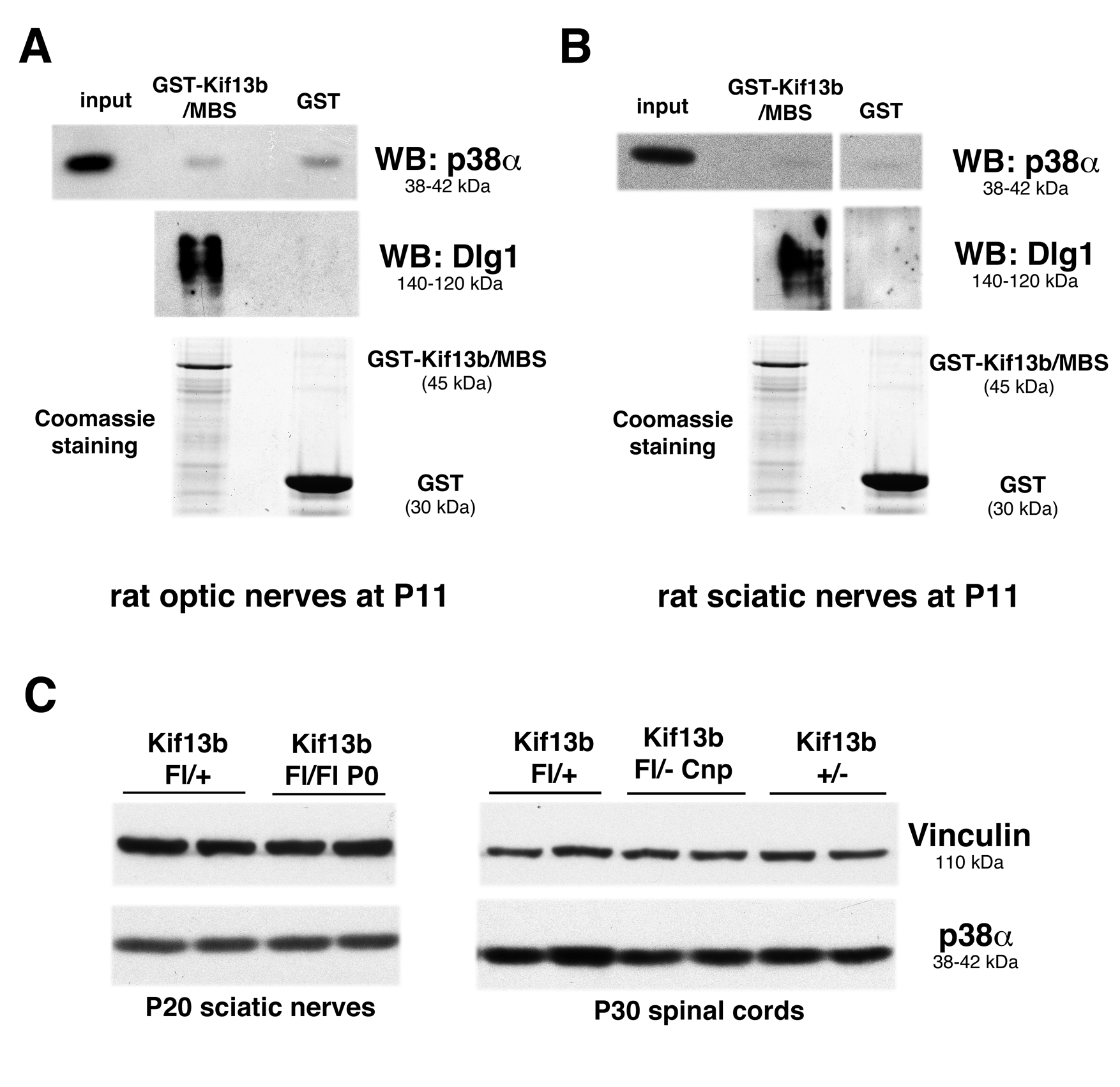

Supplement: S5 Fig — (A) GST pull down assay from P11 rat optic nerves performed using GST-Kif13b/MBS as a bait indicates that Dlg1 does not interact with p38α in rat optic nerves at P11 or (B) in sciatic nerves at P11. (C) Expression levels of p38α in both Kif13b Fl/Fl P0-Cre sciatic nerves and Kif13b Fl/Fl CNP-Cre spinal cords is similar to controls. (TIF) [file pbio.1002440.s006.tif]
